# Mutations of schizophrenia risk gene *SETD1A* dysregulate synaptic function in human neurons

**DOI:** 10.1038/s41380-025-03246-z

**Published:** 2025-09-17

**Authors:** Xiao Su, Hanwen Zhang, Yan Hong, Qian Yang, Le Wang, Tiffany Le, Jiayi Liu, Lasya Cheruvu, Emily Labour, Siwei Zhang, Karla Mendez-Maldonado, Anat Kreimer, Hongjun Song, Guo-li Ming, Jubao Duan, Zhiping P. Pang

**Affiliations:** 1 Center for NeuroMetabolism, Child Health Institute of New Jersey, Rutgers Robert Wood Johnson Medical Center, 89 French Street, New Brunswick, NJ USA; 2 Department of Neuroscience and Cell Biology, Rutgers Robert Wood Johnson Medical Center, 89 French Street, New Brunswick, NJ USA; 3 Center for Psychiatric Genetics, Endeavor Health Research Institute, Evanston, IL USA; 4 Department of Neuroscience and Mahoney Institute for Neurosciences, Perelman School of Medicine, University of Pennsylvania, Philadelphia, PA USA; 5 Department of Biochemistry and Molecular Biology, Rutgers, The State University of New Jersey, Piscataway, NJ USA; 6 Center for Advanced Biotechnology and Medicine, Rutgers, The State University of New Jersey, Piscataway, NJ USA; 7 Department of Cell and Developmental Biology, Perelman School of Medicine, University of Pennsylvania, Philadelphia, PA USA; 8 Institute for Regenerative Medicine, University of Pennsylvania, Philadelphia, PA USA; 9 The Epigenetics Institute, Perelman School of Medicine, University of Pennsylvania, Philadelphia, PA USA; 10 Department of Neurosurgery, Perelman School of Medicine, University of Pennsylvania, Philadelphia, PA USA; 11 Department of Psychiatry, Perelman School of Medicine, University of Pennsylvania, Philadelphia, PA USA; 12 Department of Psychiatry and Behavioral Neuroscience, The University of Chicago, Chicago, IL USA

**Keywords:** Neuroscience, Schizophrenia

## Abstract

Schizophrenia (SCZ) is a complex neuropsychiatric disorder associated with both common risk variants of small effect sizes and rare risk variants of high penetrance. Rare premature open reading frame (ORF) termination variants in *SETD1A* (SET Domain Containing 1A) show a strong association with SCZ; however, it remains largely unclear how rare premature ORF termination variants in *SETD1A* contribute to the pathophysiology of SCZ. To understand the impact of *SETD1A* rare premature ORF termination variants in human neurons, we CRISPR/Cas9-engineered five isogenic pairs of human induced pluripotent stem cells (iPSCs), with a recurrent heterozygous patient-specific premature ORF termination mutation c.4582-2delAG in two donor lines and a heterozygous frameshift mutation c.4596_4597insG (p. Leu1533fs) in three donor lines. These two mutations are predicted to cause a premature stop codon in exon 16 of *SETD1A*, leading to *SETD1A* haploinsufficiency. We found that these presumably loss-of-function (LoF) mutations caused the *SETD1A* mRNAs to be degraded by nonsense-mediated decay (NMD), accompanied by a reduction of full-length SETD1A protein level in iPSCs. We then characterized the morphological, electrophysiological, and transcriptomic impacts of *SETD1A*^*+/−*^ LoF mutations in iPSC-derived human excitatory neurons induced by NGN2. We found that the *SETD1A*^*+/−*^ exon-16 LoF mutations altered dendrite complexity, dysregulated synaptic transmission, and synaptic plasticity, likely by dysregulating genes involved in synaptic function. These results provide mechanistic insights into how *SETD1A*^*+/−*^ exon-16 patient-specific LoF mutations affect neuron phenotypes that may be relevant to the pathophysiology of SCZ.

## Introduction

Schizophrenia (SCZ) is a chronic and severe mental disorder with an estimated heritability of 80%, affecting 1% of the population worldwide [1]. Over the past decade, large-scale genome-wide association studies (GWAS) and exome sequencing have identified over 200 loci and around 120 risk genes associated with SCZ [2–4]. However, the mechanisms by which genetic factors contribute to SCZ pathophysiology remain largely unclear, hindering the development of mechanism-based effective therapeutics.

Compared to common GWAS risk variants with small effect sizes, rare disease risk variants with relatively larger effect sizes and high penetrance provide important insights into disease biology. *SETD1A* (SET Domain Containing 1A) is one of the strongest risk genes for SCZ [2, 5–7]. Rare SCZ risk mutations, including premature open reading frame (ORF) termination variants in *SETD1A*, are also associated with intellectual disability, autism, and other neurodevelopmental disorders (NDD) [8–12]. These rare disease-associated premature ORF termination variants mainly include nonsense (i.e., protein codon stop-gain) mutations, splice variants, and protein-coding frame-shifting small insertions/deletions (Indels), presumably leading to protein loss-of-function (LoF). *SETD1A* encodes a component of the histone methyltransferase complex that plays essential roles in producing mono-, di, and trimethylation at histone 3 lysine 4 (H3K4) specifically [13]. H3K4 trimethylation (H3K4me3) and monomethylation (H3K4me1) are canonic epigenomic marks of active gene transcriptional promoters and enhancers, respectively. Therefore, LoF in *SETD1A* may lead to an altered epigenetic landscape that causes gene expression dysregulation, contributing to the pathophysiology of SCZ. In support, histone methylation has also been suggested as one of the most enriched gene pathways in common variant-based GWAS of major psychiatric disorders [14], including SCZ [15]. Moreover, most of the identified rare neurodevelopmental and SCZ-associated mutations of *SETD1A* appeared before the region encoding evolutionarily conserved SET domain (exon 17–19), which is critical for its histone methyltransferase activity [7]. Nevertheless, it has also been reported that SETD1A has non-catalytic functions in the regulation of Cyclin-K and the DNA damage response [16], suggesting that SETD1A may function beyond an epigenetic regulator to mediate cellular function.

Haploinsufficiency in *SETD1A* has been studied in both animal and human stem cell models. Several mouse models of *Setd1a* deficiency using different strategies have been reported [17–19]. These studies suggested that neuronal morphology, synaptic transmission, and gene transcriptions are affected by *Setd1a*^*+/−*^ LoF. *Setd1a*^*+/−*^ LoF targeting exon 4 in mice shows reduced axon branches and dendritic spines, increased neuronal excitability, and increased trend of excitatory synaptic transmission, affecting short-term synaptic plasticity [17]. However, in another *Setd1a*^*+/−*^ LoF mouse model that targeted exon 7, a defective impaired excitatory synaptic neurotransmission was identified, which requires postsynaptic SETD1A to regulate the presynaptic glutamate release probability [18]. Consistent with this, mouse neurons (both excitatory and inhibitory) carrying *Setd1a*^*+/−*^ LoF by targeting exon 15 also showed a defective synaptic release [19]. Overall, these *Setd1a* haploinsufficiency mouse models suggested that synaptic transmission is likely one of the phenotypical manifestations of *Setd1a* LoF in neurons. Moreover, gene expression alteration is clearly affected by *Setd1a* LoF, likely due to altered H3K4me3 [17–19]. In humans, individuals carrying *SETD1A*^*+/−*^ LoF variants do not exhibit significant structural brain abnormalities or severe microcephaly [7, 8], which indicates that impaired proliferation and differentiation of neural progenitors are unlikely to be the major underlying causes of the disorder. Similarly, it has been shown that *Drosophila melanogaster* SETD1A orthologue is essential in postmitotic neurons for memory formation of fly brain, indicating its role in post-development neuronal function [8]. In addition to these animal models, *SETD1A*^*+/−*^ LoF is also studied in the human induced pluripotent stem cell (hiPSC)-derived neural model [20, 21]. In human-induced neuronal (iN) cells derived from one pair of isogenic hiPSCs carrying *SETD1A*^*+/−*^ LoF mutation, it was found that reduced SETD1A expression levels lead to enhanced dendritic complexity and heightened bursting activity [20]. These changes primarily manifest in glutamatergic neurons, as evidenced by altered gene sets linked to glutamatergic synaptic function in transcriptomic profiles reflecting these functional alterations [20]. In human iPSC-derived cortical spheroid models, it was reported that reduced SETD1A levels lead to reduced neurite outgrowth, spontaneous activity, and altered metabolic capacity [21]. Despite these studies, it appears that synaptic phenotypes in mouse models with *Setd1a*^*+/−*^ LoF are inconsistent with very limited numbers of human iPSC lines carrying *SETD1A*^*+/−*^ *LoF* mutations used in previous reports. Therefore, more extensive research is required to understand the synaptic and cellular phenotype in human neuronal models.

Of all the identified disease-associated *SETD1A* mutations [17–19], a two-base deletion at the exon 16 splice acceptor (c.4582-2delAG) is a recurring one that is most frequently carried by SCZ patients (four reported cases) and other NDD patients (three incidences) [8]. The splice variant c.4582-2delAG is predicted to retain the intron 15, which leads to a different reading frame [7]. To understand the impact of patient-specific mutation of *SETD1A* in a human context, we CRISPR/Cas9-engineered five isogenic pairs of iPSC lines carrying c.4582-2delAG or a frameshift mutation c.4596_4597insG (p. Leu1533fs) that mimics the LoF of this patient-specific mutation. We generated excitatory human induced neurons (iNs) by ectopic expression of Ngn2 [22] and comprehensively characterized the transcriptional, morphological, and functional impacts of the patient-specific mutation in Ngn2-iNs. Overall, our results provide compelling evidence that supports *SETD1A*^*+/−*^ exon 16 LoF mutations leading to nonsense-mediated decay (NMD) of *SETD1A* mRNA, altered neuronal dendrite complexity, and dysregulated synaptic function through transcriptional modifications.

## Materials and methods

### Induced pluripotent stem cells (iPSCs)

Four iPSC lines (CD07, CD15 and CD19, and C11C) were selected for CRSIPR/Cas9 editing. Human iPSC lines (CD07 (male), CD15 (female), and CD19 (male)) were obtained from RUCDR NIMH Stem Cell Center (www.nimhgenetics.org). These iPSC lines were generated from cryopreserved lymphoblasts deposited by the Molecular Genetics of Schizophrenia (MGS) consortium [23], which had collected the biomaterials with informed consent as approved by the Endeavor Health (formerly NorthShore University HealthSystem) institutional review board (IRB). All donors are controls of European ancestry without large SZ-risk CNVs from the MGS study [23]. As part of previous studies [24, 25], the MGS donor-derived iPSC lines have been characterized for pluripotency by immunofluorescence (IF) staining of pluripotency markers and by RNA-seq-based Pluritest [26]. The C11C iPSC line was derived from repository male dermal fibroblasts of a Caucasian donor obtained from the Lieber Institute for Brain Development, which has been extensively characterized by a previous publication [27]. The absence of chromosomal abnormality of these iPSC lines was confirmed by RNA-seq-based eSNP-Karyotyping [24, 25].

All iPSCs were cultured at 37 °C, 5% CO_2_ on Matrigel® Matrix (Corning Life Sciences, CB-40234)-coated plates in mTeSR™ Plus medium (Stem Cell Technologies, # 100-0276) and passaged every 3–4 days using Accutase (Stem Cell Technologies, # 07920). The detailed procedure can be found in previous studies [28].

### CRISPR editing of *SETD1A* in iPSCs

gRNA and ssODN for *SETD1A* editing were designed, and gRNA was cloned into pSpCas9(BB)-2A-Puro (PX459) V2.0 vector (Addgene, # 62988) following a previously published protocol [29]. For editing, 24 h before transfection, a 60–70% confluent iPSC culture was dissociated using Accutase (Stem Cell Technologies, # 07920) and replated onto matrigel-coated 60 mm dish at 4.5 × 10^5^ per well in mTeSR™ Plus with 5 µM ROCK inhibitor (R&D systems, # 1254/1). The next day, DNA was transfected using LipofectamineSTEM (Thermofisher, # STEM00001) at 1:2 DNA: reagent ratio following vendor’s instructions in antibiotics-free mTeSR™ Plus with 5 µM ROCK inhibitor. For the exon-16 1 bp insertion editing, 4 µg/well of Cas9 plasmid carrying the gRNA was introduced; for patient-specific 2 bp deletion editing, 3 µg/well of Cas9 plasmid carrying the gRNA and 3 µg ssODN were used. 24 h post transfection, cells were selected in mTeSR™ Plus with 0.5 µg/ml puromycin. The media was refreshed with 0.25 µg/ml puromycin 48 h post-transfection. 72 h post-transfection, the selection was withdrawn, and cells were maintained in regular mTeSR™ Plus media for 7–10 days until colonies appeared and were large enough for genotyping. Colonies were manually picked onto 96-well plate and the genotypes at the mutagenesis site were confirmed by Sanger sequencing. All edited clones were confirmed by Sanger sequencing to be free of off-target editing at five top-ranking predicted off-target sites. The gRNA, ssODN, and primer sequences are available in Supplementary Table 1.

The guide RNA was automatically generated by the online tool/software (www.benchling.com) or online tool CRISPick (https://portals.broadinstitute.org/gppx/crispick/public). The off-targets loci were predicted by the online tool Off-Spotter (https://cm.jefferson.edu/Off-Spotter/) when we designed the on-Target gRNA. The gRNAs in this off-target output file usually have 3–4 mismatches, and the software would calculate a score for each and rank them. The top 5 predicted off-target loci were checked by PCR and Sanger sequencing.

### Lentivirus production

Lentiviruses were produced as described [30] in HEK 293FT cells (ATCC) through co-transfection with 3rd generation helper lentivirus plasmid (pMDLg/pRRE, VsVG and pRSV-REV) using calcium phosphate transfections. Lentiviral particles were collected in mTeSR™ Plus medium, aliquoted, and stored at −80 °C. The following lentivirus constructs were used: (1) FUW-TetO-Ngn2-P2A-puromycin; (2) FUW-rtTA; (3) FSW-Venus; (4) pMax-GFP.

### Generation of iN Cells from iPSCs

Excitatory- (i.e. Ngn2-iNs) were generated as described [22]. Briefly, iPSCs were dissociated by Accutase (Stem Cell Technologies, # 07920) and plated on six-well plate coated with Matrigel® Matrix (Corning Life Sciences, CB-40234) and mTeSR™ Plus medium containing RHO/ROCK pathway inhibitor Y-27632 (Stemgent, # 04-0012-02) or CEPT including Chroman 1 (MedChem Express, HY-5392), Emricasan (SelleckChem, S7775), Polyamine supplement (Sigma-Aldrich, P8483), and Trans-ISRIB (R&D Systems, 5284). At the same time of the plating, lentivirus cocktails prepared as described above (200 μl of Ngn2-contained virus and 200 μl of rtTA-contained virus per well of six-well plate) were added. On day 1, the culture medium was replaced with Neurobasal™ Medium (Gibco™, 21103049) containing doxycycline (MP Biomedicals, # 24390-14-5, 2 μg/ml), and doxycycline was maintained in the media for ~10 days. On day 2 and 3, infected cells were selected by puromycin (Sigma-Aldrich, P8833, 1 μg/ml). On day 4, mouse glia cells were plated on Matrigel-coated coverslips (5 × 10^4^ cells/single well of 24 well plates), and the culture medium was replaced with Neurobasal™ Medium containing doxycycline (2 μg/ml) for recovery. On day 5, Ngn2-iNs (total of 2–3 × 10^5^ cells/well) were plated on a monolayer of mouse glial cells. On Day 6, media change occurred using Neurobasal medium with 5% FBS (fetal bovine serum, R&D Systems S11550) containing growth factors 10 ng/ml of BDNF (Peprotech, 450-02), GDNF (Peprotech, 450-10), NT3 (Peprotech, 450-03), B-27™ Supplement (Gibco™, 17504044), 1% GlutaMAX™ (Gibco™, 35050061), and Cytosine β-D-arabinofuranoside (AraC, Sigma-Aldrich, C1768, 2 μM), which was added to stop glial cell division. Morphological, biochemical, and functional analyses were conducted at 5 to 6-week-old. Each of the morphological and functional analyses was conducted from at least two or three independent culture batches for each isogenic pair.

### Mouse glial culture

Mouse glial cells were used to facilitate the human neuronal growth and synapse formation, as described previously [22]. Wild-type C57BL/6J mice at postnatal day 0–3 (P0–3) were used for glial culture. Briefly, P0-P3 mouse cortices were digested with papain, 1 μM Ca^2+^, and 0.5 μM EDTA solution for 15 min at 37 °C. Glial cells were passaged twice to eliminate mouse neuron cells and cultured with 10% fetal bovine serum in DMEM (Invitrogen). All animal work was performed without gender bias under the Institutional Animal Care and Use Committee (IACUC) protocol approved by the Rutgers University IACUC Committee.

### Immunohistochemistry

Primary antibodies were diluted in blocking buffer (1% goat serum (Gibco™, 16210072), 0.25% Triton X-100 and 4% BSA) at 4 °C and incubated overnight. Coverslips were washed 3 times with blocking buffer and then incubated with secondary antibody (1: 500) at RT for 1 h. Following three washes with PBS, coverslips were mounted onto glass microscope slides with mounting medium containing DAPI (Sigma-Aldrich, F6057). The following antibodies were used: SOX2 (Millipore Sigma AB5603, 1:1000), Oct4 (Millipore Sigma MAB4401/4419, 1:1000), Tra-1-60 (Millipore Sigma MAB4360, 1:1000), MAP2 (Sigma-Aldrich M1406, 1:1000; Millipore Sigma AB5543,1:1000), Synapsin-1 (E028, 1:3000; SYSY 106011,1:1000). At least two-three independent culture batches for each isogenic pair were used for all experiments.

### Sparse transfection in iN cells

To quantify changes in neurite outgrowth, Ngn2-iNs were sparsely transfected with FSW-Venus or pMax-GFP construct at day 40 using calcium phosphate-based transfection. Briefly, per one well of a 24-well format, 0.5 µg of GFP plasmid, 2.5 M CaCl_2_, and molecular-grade water were mixed and added dropwise to 2X HBS (280 mM NaCl, 1.5 mM Na_2_HPO_4_, 50 mM HEPES) on a gentle vortex, and the transfection mix was incubated at room temperature for 10 min. After washing wells three times with MEM+++ (MEM containing 0.5 mM CaCl_2_, 1 mM MgCl_2_, and 4% sucrose to minimize coverslip peeling), the transfection mix was added dropwise to each well and incubated for 30 min at 37 °C. Wells were washed three times with MEM, and transfection efficiency was confirmed 48–72 h later.

### Image acquisition and quantification of neurite outgrowth

All images were taken with a Zeiss LSM700 laser-scanning confocal microscope. Neurite outgrowth was captured using a 20× objective at 512 × 512-pixel resolution with tile scan and Z-stack function. Specifically, the filamenting modules of Imaris software (Bitplane version 9.5) were used to quantify changes in neurite outgrowth (metrics: number of branch points, total neurite length and *Sholl* analysis). Statistical analysis was performed using Mann-Whitney test (bar graphs) and two-way ANOVA (Sholl analysis) comparing control to each individual mutation (*p 0.05, **p 0.01, ***p 0.001; nonsignificant comparisons were not indicated).

### Synaptic puncta density analysis

Immunofluorescence was performed on iNs at day 35–42 with dendrite marker MAP2 and synaptic marker Synapsin-1. Synapse formation was imaged using a Zeiss LSM700 laser-scanning confocal microscope with a 63× objective at 1024 × 1024 pixel resolution. All images were acquired using Z-stack maximal projection in Zeiss Zen Blue software. Correlated synaptic puncta number was quantified by Intellicount as reported previously [31]. Primary processes were blindly counted and quantified.

### Western blot analysis

For iPSCs culture on 6-well plate, the cultures were washed twice with ice-cold PBS and then the cells were lysed with 100 μL RIPA buffer (50 mM Tris-HCl, pH7.5, 150 mM NaCl, 1% NP-40 (Igepal CA-630), 0.5% Sodium deoxycholate, 0.1% SDS, 1 mM DTT, 1X Protease Inhibitor cocktail and phosphatase inhibitor) for each well. The protein concentration was quantified by BSA kit (Thermo Scientific, #23227). For induced neuron culture on coverslips, at day 35–42, the cultures were washed once with ice-cold PBS. The proteins were directly collected by 2X SDS loading buffer (4% SDS, 125 mM Tris HCl (pH 6.8), 20% glycerol, 5% 2-mercaptoethanol, and 0.01% bromophenol blue) [32]. Protein samples were then denatured by heating to 95 °C for 5 min. The denatured protein samples were allowed to cool down, and equal amounts of the samples were analyzed by 4–15% or 7.5% TGX gels (Bio-Rad #4561084DC, #4561023DC) for sequential western blots on the same membranes. The protein from the gel was transferred to a 0.45 or 0.22 μm nitrocellulose membrane (Bio-Rad #1620115) using the Bio-Rad transferring system. The nitrocellulose membrane was blocked in 10% nonfat milk for 1 h at room temperature. After the blocking step, membranes were incubated with primary antibody overnight at 4 °C. The following day, the blots were washed three times with Tris-buffered saline with 0.1% Tween 20 (TBST) before adding horseradish peroxidase-conjugated secondary antibody at room temperature for 1 h. Primary antibodies catalog number (N-SETD1A Invitrogen PA5-35975; SETD1A Abcam AB243881; H3K4me3 Cell Signaling 9751S; H3 Sigma-Aldrich H0164; VCP K330). Protein bands were visualized by the addition of Clarity Western ECL Substrate (Bio-Rad #1705060). The density of the bands was quantified using ImageJ software. Beta-actin was measured as a loading control.

### Electrophysiological recordings

Whole-cell patch-clamp electrophysiology for Ngn2-iNs was performed as described [28]. For spontaneous postsynaptic current and current-clamp recordings, K-gluconate internal solution was used, which consisted of (in mM): 126 K-gluconate, 4 KCl, 10 HEPES, 0.05 EGTA, 4 ATP-magnesium, 0.3 GTP-sodium, and 10 phosphocreatine. For miniature postsynaptic current recording, a Cs-based internal solution was used, which consisted of (in mM): 40 CsCl, 3.5 KCl, 10 HEPES, 0.05 EGTA, 90 K-gluconate, 1.8 NaCl, 1.7 MgCl_2_, 2 ATP-magnesium, 0.4 GTP-sodium, and 10 phosphocreatine. Miniature excitatory postsynaptic currents (mEPSCs) were observed for iN cells in voltage-clamp mode with a holding potential of -70 mV in the presence of 1 μM tetrodotoxin (TTX). For evoked recording, a Cs-based internal solution was used with QX314 (5 mM). The bath solution contained (in mM): 140 NaCl, 5 KCl, 10 HEPES, 2 CaCl_2_, 2 MgCl_2_, 10 Glucose, pH 7.4. To elicit action potentials, a series of increasing amounts of current was applied to the cell in 10-pA increments across 19 independent sweeps (−50 pA to +130 pA). Each step lasted for 1 s, and action potential kinetics were analyzed in Clampfit (11.1) for the first triggered action potential observed at the lowest state of the current injection. All cell culture recordings were conducted in the whole-cell configuration at room temperature.

### RNA isolation and RNA sequencing

For qPCR reactions, cells from hiPSCs and iNs cultures were lysed in RLT plus buffer, and total RNA was purified with the RNeasy mini-Kit (QIAGEN). For RNA sequencing, the cultured iNs were lysed in RLT plus buffer (QIAGEN) and subjected to RNA sequencing (RNA-seq) at GENEWIZ from Azenta (South Plainfield, NJ) and Novogene (Sacramento, CA).

### Quantitative RT-PCR

Purified RNA was eluted in 35 uL per sample. Purified total RNA was reverse-transcribed and PCR-amplified using cDNA reverse transcription kit (Invitrogen #11755050). The qPCR reactions were conducted with PowerUp SYBR Green Master Mix for qPCR kit (Applied Biosystems, # A25742). The mRNA levels were quantified by real-time PCR assay using the QuantStudio 3 System and RQ analysis software (Thermo Fisher Scientific). Within each plate, the housekeeping gene GAPDH was included as an internal control. The SETD1A primers are listed in Supplementary Table 2.

### Gene expression analyses

#### Bulk RNA-sequencing

For samples with c.4596_4597insG mutation, RNA-seq files were provided in the format of 2 × 150 bp paired-end fastq files from GENEWIZ from Azenta. Briefly, cDNA libraries were prepared with the polyA selection method with Illumina HiSeq kit and customized adapters. 20–30 M paired-reads were recovered from each sample at the rate of ∼350 M raw read-pairs per lane on an Illumina HiSeq 2000. No Trimmomatic-based adapter-trimming was performed, as the sequencing facility had performed pre-cleaning on raw reads. Raw files were subsequently mapped to the human hg38 genome (GRCh38p7.13) with STAR v2.7.0 and GENCODE v35 annotation file.

For samples with c.4582-2delAG mutation, paired-end reads per sample were collected using the NovaSeq PE150 platform (Novogene) with polyA enrichment. Quantified libraries were pooled and sequenced on Illumina NextSeq500 platforms by Novogene. Approximately 40–60 million paired reads were generated for each sample. Reads were aligned to the human hg38 reference (GRCh38p7.13) genome by HISAT2 (v.2.1.0) [33].

#### Differential gene expression analysis

DESeq2 (v1.28.1) was used to perform DEG analysis for raw count values of samples with the design formula as “design = ~ cell_lines + genotypes”, with “cell_lines” as the key of paired samples and “genotypes” as the key of genotype annotations. Significance test results from DESeq2, including log2 fold changes and FDR-adjusted p-values, were used for downstream analysis. Ensembl ids were transferred to gene symbols using the biomaRt package (v2.44.4) with Ensembl as the host. We used our 2 DEG lists (c.4596_4597insG vs. WT; c.4582-2delAG vs. WT) and investigated overlapping genes (up/down/both) separately. Genes with adjusted p values < 0.05 and absolute log_2_FC > 0 were called DEGs.

### SynGo annotation

For analysis of DEGs associated with synaptic function, GO analysis was performed using the SynGO [55] website: https://www.syngoportal.org/.

### WGCNA

Gene counts were normalized using ComBat seq [34] to remove the batch effect. The network was constructed using the WGCNA package (v1.70.3) [35] through a signed network. A soft-threshold power of 18 was used to achieve approximate scale-free topology (R^2^ > 0.8). Module GO term enrichment was performed using cluster Profiler (v4.0.5) [36] with default parameters. The module network is visualized using Cytoscape (v 3.10.2) [37].

### eSNP-Karyotyping analysis

#### RNA-Seq data processing

RNA-Seq data were initially processed using the nf-core/rnaseq pipeline (version 3.12.0) executed via Nextflow (version 23.04.4) [38]. In detail, we merged the raw FastQ files using the *cat* (version 8.30) command to consolidate data from technical replicates. Next, we auto-inferred the strandedness of the libraries through subsampling and pseudo-alignment using *fq* (version 0.9.1 (2022-02-22) and *Salmon* (version 1.10.1) [39]. Then, we conducted quality control checks with FastQC (version 0.11.9) and trimmed adapters and low-quality sequences using Trim Galore! (version 0.6.7).

After we obtained the trimmed reads, we combined trimmed paired-end reads and aligned them to the human reference genome (GRCh38) using the *Bowtie2* aligner (version 2.4.1), resulting in a set of Sequence Alignment/Map (SAM) format files [Bowtie2]. We then converted the SAM file to binary alignment and map (BAM) format for downstream processing using *Samtools* View (version 1.3.1) with the “-bS” flag enabled [40].

#### Cell line karyotyping

First, we called variants from pre-processed BAM files against the reference genome using the GPU-accelerated *haplotypecaller* available with the *NVIDIA Parabricks toolkit* (version 4.3.1, https://docs.nvidia.com/clara/parabricks/4.3.1/).

We then assessed the potential presence of chromosomal multiplications using *eSNP-karyotyping* method [41], analyzing the binary alignment map (BAM) and VCF files in conjunction with dbSNP (version 155) [42]. Following previously published methods, we compared the allelic ratio in sliding windows of 151 single-nucleotide polymorphisms (SNPs) with a minimal allele frequency above 0.2 and were covered by more than 20 reads with the rest of the genome. We identified significant multiplications as those with a false discovery rate below 0.05.

### Quantification and statistical analysis

Statistical analysis was performed using GraphPad Prism 10.0. All the data is presented as mean ± standard error of the mean (SEM), unless otherwise noted. All experiments were repeated on at least two-three biological replicates for each isogenic pair, with each replicate consisting of an independent infection/differentiation. Data were tested for normality using the Shapiro-Wilk test. Statistical significance (*p 0.05, **p 0.01; ***p 0.001) was evaluated with the Kolmogorov-Smirnov test (for cumulative probability plots), two-way ANOVA, unpaired Student’s t-test and Mann-Whitney test.

## Results

### Generation of a *SETD1A*^*+/−*^ LoF iPSC lines mimicking patient with *SETD1A* haploinsufficiency (c.4582-2delAG and c.4596_4597insG (p. Leu1533fs))

**Fig. 1:**
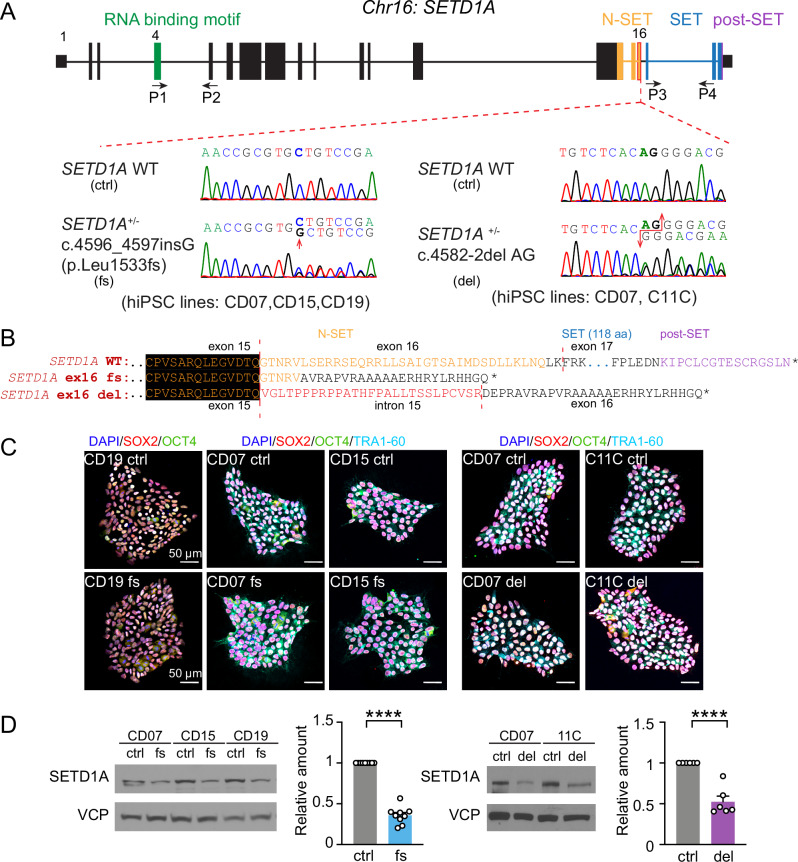
Generation and validation of iPSCs carrying *SETD1A*^*+/−*^*LoF* mutations. **A** Diagram showing the genomic structure of *SETD1A* and the two sets of mutations. Two sets of *SETD1A*^*+/−*^ LoF mutations (c.4596_4597insG (p.Leu1533fs) and c.4582-2del AG) in four different genetic backgrounds (CD07, CD15, CD19, C11C) were generated. *Low panels:* Sanger sequencing confirms the mutations. **B** The protein sequence of SETD1A and predicted protein sequence of *SETD1A*^*+/−*^ LoF mutations. **C** Representative images of iPSCs with pluripotency markers SOX2, OCT4 and TRA1-60 and nucleus marker DAPI. Scale bars: 50 μm. **D** Western blot of SETD1A protein normalized to VCP in iPSCs. *N* = 9 for c.4596_4597insG mutation (3 independent batches of 3 isogenic pairs) and *N* = 4 for c.4582-2del AG mutation (2 independent batches of 2 isogenic pairs). Statistical significance was assessed by unpaired t-test. Data are shown as means ± SEM. *P < 0.05, **P < 0.01, ***P < 0.001 and **** *P* < 0.0001.

To study patient-specific mutations in an iPSC-derived human neural model, we generated five pairs of isogenic *SETD1A*^*+/−*^ LoF mutation iPSC lines using the CRISPR-Cas9 gene editing technology (Fig. 1A). First, we generated a patient-specific knock-in mutation c.4582-2delAG by targeting the Intron 15-Exon 16 boundary of *SETD1A* in two healthy control iPSC lines (CD07 and C11C). Meanwhile, we also created an exon 16 frameshift (fs) mutation by generating a 1-base pair (bp)-insertion mutation c.4596_4597insG (p. Leu1533fs) in three healthy control iPSC lines (CD07, CD15, CD19) to mimic *SETD1A*^*+/−*^ LoF caused by c.4582-2delAG. The mutated DNA sequences were confirmed by Sanger sequencing (Fig. 1A), and no off-targets were found for the top 5 predicted loci (Supplementary Table 1). The c.4582-2delAG in *SETD1A* disrupted the splicing adaptor sequences and retained the intron 15, which led to a short fragment of de novo protein sequence after exon 15 and a premature stop codon in exon 16 (Fig. 1A, B). The c.4596_4597insG in exon 16 led to a premature stop codon and thus, protein truncation. Both mutations led to premature stop codons in one chromosome and led to *SETD1A* haploinsufficiency. These gene targeting strategies did not yield iPSC lines carrying homozygous *SETD1A* mutations, which is consistent with the literature that shows *SETD1A* homozygous knockouts in germline cells as lethal [43]. As a result, five pairs of isogenic heterozygous *SETD1A* LoF mutation iPSCs were generated to model humans with heterozygous *SETD1A* LoF alleles. The pluripotency of all generated iPSC lines was confirmed through immunostaining with pluripotency markers OCT4, SOX2, and Tra1-60 (Fig. 1C and S1).

We next examined the expression of SETD1A protein to confirm the LoF premature ORF frame shift stop codon-mutations in the *SETD1A* gene using Western blotting. Consistent with the reported data from the isolated patients’ lymphoblastoid cell lines carrying the same mutation (c.4582-2delAG) [8], we observed a significant decrease of full-length SETD1A protein levels in iPSCs carrying c.4582-2delAG and c.4596_4597insG mutations through two sets of SETD1A antibodies that target either exon 2 or exon 14 of *SETD1A*, respectively (Figs. 1D, S2A).

### Mutant *SETD1A* mRNAs are degraded through nonsense-mediated decay (NMD)

**Fig. 2:**
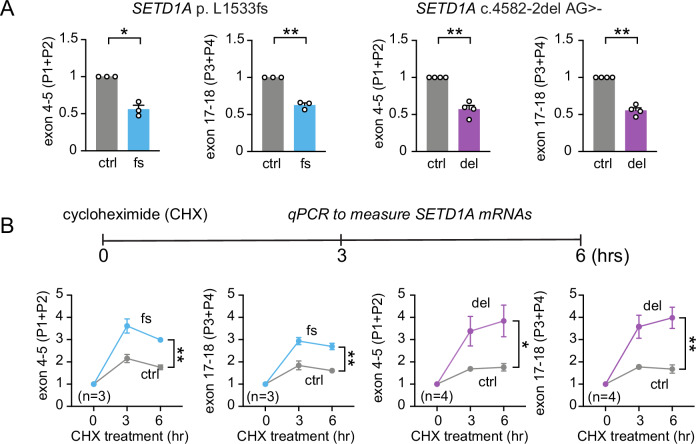
Down-regulation of mRNAs in iPSCs carrying *SETD1A*^*+/−*^ LoF is mediated by NMD. **A** Normalized *SETD1A* mRNA expression in *SETD1A*^*+/−*^ LoF iPSCs using two sets of primer as depicted in Fig. 1A: P1 + P2 for exon 4–5 and P3 + P4 for exon 17–18. **B** Impact of cycloheximide (CHX) exposure (0, 3, 6 h) on *SETD1A* mRNA expression levels in iPSCs carrying *SETD1A*^*+/−*^ LoF. N = 3 for c.4596_4597insG mutation (1 batch of 3 isogenic pairs) and N = 4 for c.4582-2del AG mutation (2 batches of 2 isogenic pairs). Statistical significance was assessed by t-test for qPCR in(**A**) and two-way ANOVA for qPCR after CHX treatment in (**B**). Data are shown as means ± SEM. *P < 0.05, **P < 0.01, ***P < 0.001 and **** *P* < 0.0001.

Having demonstrated that iPSCs carrying c.4582-2delAG and c.4596_4597insG mutations cause a reduction in SETD1A protein expression (Figs. 1D, S2A), we hypothesize that mutant *SETD1A* mRNAs are degraded. Thus, we quantified the *SETD1A* mRNA level and tested the mechanisms that regulate *SETD1A* mutant mRNA degradation. It is known that NMD is a translation-dependent mRNA surveillance pathway that reduces errors in gene expression by eliminating mRNA transcripts that contain premature stop codons [44]. Although the SCZ patient-specific mutations c.4582-2delAG and c.4596_4597insG are both predicted to generate premature ORF termination in exon 16, it is unknown whether these mutations indeed cause NMD of *SETD1A* mRNA. Towards this end, we first tested whether the mRNA was degraded by using two different sets of primers that target different transcript regions before or after the CRISPR-introduced mutations (P1 + P2 for exons 4–5 and P3 + P4 for exons 17–18). Consistent with the ~50% reduction of full-length SETD1A protein level in iPSCs (Fig. 1D), we found ~50% reduction of *SETD1A* mRNA level in iPSCs when interrogating the two different transcript regions (Fig. 2A), suggesting NMD may occur for the mRNAs produced by the LoF alleles (c.4582-2delAG and c.4596_4597insG).

To further corroborate whether these *SETD1A* mutations indeed lead to NMD of the mRNA, we treated iPSCs with a translation inhibitor cycloheximide (CHX,100ug/ml) for 3 and 6 h and then examined the expression level of *SETD1A* mRNA. CHX inhibits protein translation and subsequently interferes with the NMD machinery’s recognition of a premature stop codon. We observed that *SETD1A* transcripts increased dramatically after CHX treatment in iPSC lines carrying c.4596_4597insG or c.4582-2delAG mutation when compared with control wild-type (WT) iPSCs (Fig. 2B). These results indicate that both patient-specific mutations c.4582-2delAG and c.4596_4597insG in exon 16 of *SETD1A* cause protein LoF through NMD of *SETD1A* mRNAs.

### *SETD1A*^*+/−*^ LoF excitatory Ngn2-iNs showed decreased dendrite complexity

**Fig. 3:**
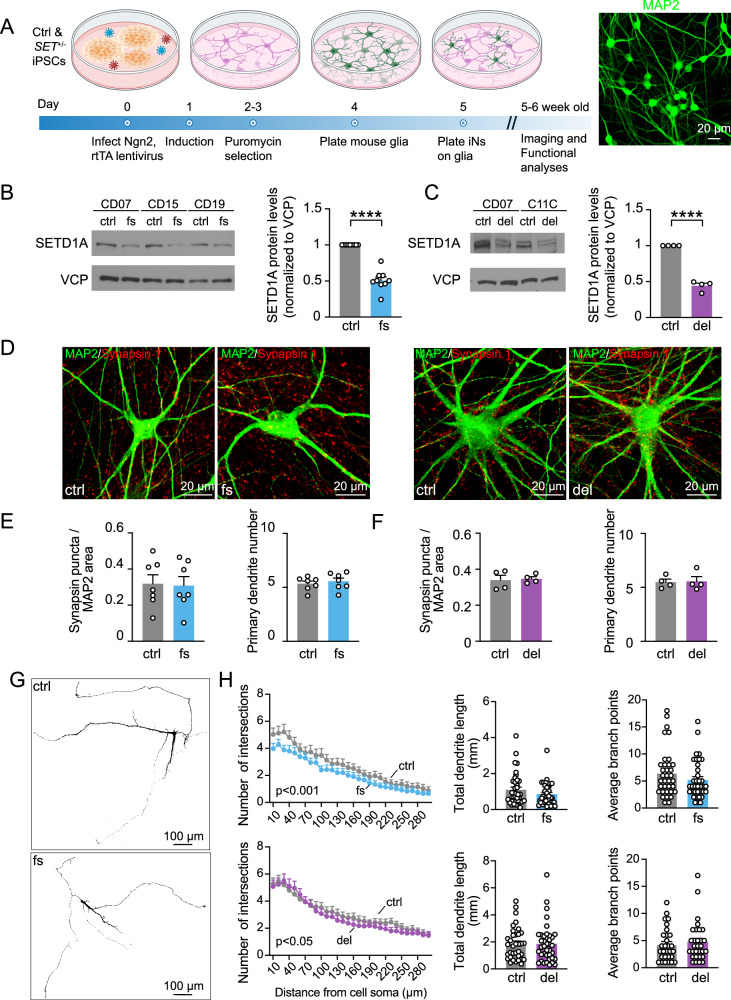
Morphological characterization of Ngn2-iNs carrying *SETD1A*^*+/−*^ LoF mutations. **A** Diagram showing the experimental design and the timeline of Ngn2-iNs culture. **B**, **C** Western blot of normalized SETD1A protein expression in Ngn2-iNs for c.4596_4597insG and c.4582-2delAG mutations, respectively. N = 9 for c.4596_4597insG mutation (3 independent batches of 3 isogenic pairs) and N = 4 for c.4582-2del AG mutation (2 independent batches of 2 isogenic pairs). **D** Representative images of Ngn2-iNs with IHCs of neuronal marker MAP2 and pre-synaptic marker synapsin-1 in control and *SETD1A*^*+/−*^ mutant neurons. **E**, **F** Quantification of synaptic puncta number normalized to MAP2-labelled dendrites and the number of primary dendrites for c.4596_4597insG and c.4582-2del AG mutations, respectively. Each dot represents the average of one batch of one isogenic pair. N = 7 for c.4596_4597insG mutation (3 batches of 1 isogenic pair and 2 batches of 2 isogenic pairs). N = 4 for c.4582-2delAG mutations (2 batches of 2 isogenic pairs). **G** Representative images of neurons sparsely labeled by GFP for dendrite morphology analysis. **H** Quantification of neuronal dendrite morphology using *Sholl* analysis. Total dendrite length and average branch points were quantified. Each dot represents one neuron in bar graph. N = 34 for control and N = 36 for c.4596_4597insG from 3 independent batches. N = 35 for control and N = 38 for c.4582-2delAG from 3 independent batches. Statistical significance was assessed by Mann-Whitney test (bar graphs) and two-way ANOVA (*Sholl* analysis). Data are shown as means ± SEM. *P < 0.05, **P < 0.01, ***P < 0.001 and **** P < 0.0001.

To investigate the impact of *SETD1A*^*+/−*^ LoF on the human neural model, we generated excitatory Ngn2-iNs (Fig. 3A) using isogenic control and mutant iPSCs. These iNs were replated on monolayer mouse astrocytes to facilitate their maturation and synapse formation [45]. Mutant iPSCs carrying *SETD1A*^*+/−*^ LoF mutations, i.e., c.4582-2delAG or c.4596_4597insG, could produce mature Ngn2-iN cells similar to control iPSCs. We next examined the expression of SETD1A proteins by Western blotting. Similar to that found in iPSC lines, we observed ~50% reduction of full-length SETD1A proteins when compared with control neurons (Fig. 3B, C).

We next examined the impact of *SETD1A*^*+/−*^ LoF mutations on synaptogenesis. We conducted immunostaining with dendritic marker MAP2 and presynaptic marker synapsin-1 (Fig. 3D) for 5 to 6-week-old excitatory human iNs. We used a custom-made algorithm, *Intellicount* [31], to unbiasedly quantify synapsin-1 labeled synaptic puncta colocalized with MAP2-labelled dendrites and found no significant differences between control and mutant neurons (Fig. 3E, F).

To further characterize the impact of *SETD1A*^*+/−*^ mutations on the morphogenesis of human neurons, particularly the neuronal dendrite development, we sparsely labeled neurons with pMax-GFP. We reconstructed neuronal 3D structure to analyze the dendrite complexity and length (Fig. 3G). We found that mutant neurons carrying the c.4582-2delAG and c.4596_4597insG mutations displayed reduced dendrite complexity through *Sholl* analysis without affecting the dendrite length and number of branch points (Fig. 3H).

### *SETD1A*^*+/−*^ LoF alters synaptic transmission and short-term synaptic plasticity in Ngn2-iN cells

To investigate the impact of *SETD1A*^*+/−*^ LoF on neuronal functions, including intrinsic electrophysiological properties and synaptic transmission, we performed whole-cell patch clamp recordings from 5 to 6-week-old excitatory human iNs cultured on monolayer astrocytes. We first measured intrinsic electrophysiological parameters, such as cell membrane capacitance and resistance (Fig. S3A–D). We observed increased membrane capacitance and decreased membrane resistance for c.4596_4597insG but no significant differences for c.4582-2delAG. Next, we characterized the neuronal excitability and analyzed the action potential (AP) kinetics (Fig. S4). We found that neurons carrying c.4582-2delAG mutation showed increased neuronal excitability without affecting the kinetics of APs, including AP threshold, amplitude, afterhyperpolarization potential (AHP), and half-width of APs.

**Fig. 4:**
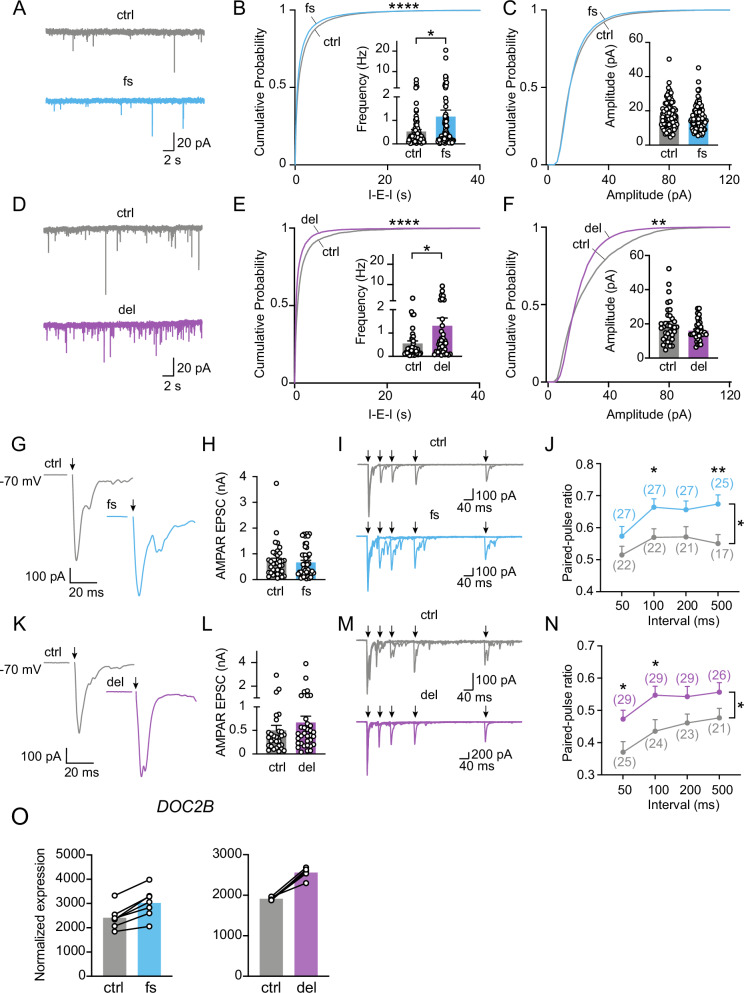
Functional characterization of Ngn2-iNs carrying *SETD1A*^*+/−*^ LoF mutations. **A** Representative traces of mEPSC recording for control and c.4596_4597insG mutant Ngn2-iNs. **B**, **C** Cumulative distribution and quantification of mEPSC frequency (inter-event-interval, I-E-I) in (**B**) and mEPSC amplitude in (**C**). **D** Representative traces of mEPSC recording for control and c.4582-2delAG mutant Ngn2-iNs. **E**, **F** Cumulative distribution and quantification of mEPSC frequency (inter-event-interval, I-E-I) in (**E**) and mEPSC amplitude in (**F**). **G** Representative traces of evoked AMPAR EPSC for control and c.4596_4597insG mutant Ngn2-iNs. **H** Quantification of evoked AMPAR EPSC amplitude. **I** Representative traces of paired-pulse recording traces for control and c.4596_4597insG mutant Ngn2-iNs. **J** Quantification of paired-pulse ratio. **K** Representative traces of evoked AMPAR EPSC for control and c.4582-2delAG mutant Ngn2-iNs. **L** Quantification of evoked AMPAR EPSC amplitude. **M** Representative traces of paired-pulse recording traces for control and c.4582-2delAG mutant Ngn2-iNs. **N** Quantification of paired-pulse ratio (PPR). **O** Normalized expression of DOC2B in RNAseq data for c.4596_4597insG and c.4582-2delAG mutations. Statistical significance was evaluated with the Kolmogorov–Smirnov test (cumulative probability plots), Mann-Whitney test (frequency and amplitude) and two-way ANOVA (paired-pulse ratio plots). Each stimulation interval was also evaluated by Student’s t test (paired-pulse ratio plots). *P < 0.05, **P < 0.01, ***P < 0.001 and **** P < 0.0001.

Synaptic transmission is vital for neuronal communication and signal transmission. Next, we asked whether *SETD1A*^*+/−*^ LoF mutations alter synaptic function in human iNs. We first analyzed miniature excitatory postsynaptic currents (mEPSCs) (Fig. 4). We found that mutant iN cells showed higher mEPSC frequency for both c.4596_4597insG and c.4582-2delAG mutations (Fig. 4B, E). However, we did not observe significant differences for mEPSC amplitude except for altered cumulative probability distribution of amplitudes in neurons carrying c.4582-2delAG mutation (Fig. 4C, F). These data suggest that the overall spontaneous miniature synaptic transmission is enhanced in human neuronal cells carrying *SETD1A*^*+/−*^ LoF mutations.

To further investigate the synaptic alterations by *SETD1A*^*+/−*^ LoF mutations, we recorded evoked EPSCs (eEPSCs) by an extracellular stimulation electrode [46] in mutant *SETD1A*^*+/−*^ LoF iNs. We did not observe changes in evoked EPSCs amplitude in either c.4596_4597insG or c.4582-2delAG mutant *SETD1A*^*+/−*^ LoF iNs (Fig. 4G, K). We then assayed the short-term synaptic plasticity using a paired-pulse protocol with intervals between 50 to 500 ms. Because of no apparent differences in the sizes of eEPSCs between the control and the mutant iNs, we were expecting no change in the paired-pulse ratio (PPR, eEPSC2/eEPSC1). To our surprise, we observed a paradoxical, but consistent, increase in the PPRs, suggesting an alteration in short-term synaptic plasticity (recovering or replenishment of readily releasable pool of synaptic vesicles) in neurons carrying *SETD1A*^*+/−*^ LoF mutations (Fig. 4I–N).

Interestingly, in our RNA seq data, the expression levels of presynaptic gene *DOC2B* (Fig. 4O), which is involved in regulating spontaneous synaptic releases [47], are significantly increased in iNs carrying both c.4596_4597insG and c.4582-2delAG mutations. This may contribute to the increased spontaneous release in SETD1A LoF mutant Ngn2-iNs.

Collectively, our electrophysiological data suggest that neurons with patient-specific *SETD1A*^*+/−*^ LoF mutations c.4582-2delAG and c.4596_4597insG exhibited altered neuronal excitability, dysregulated synaptic transmission and short-term synaptic plasticity.

### Transcriptional profiling characterization of *SETD1A*^*+/−*^ LoF Ngn2 iNs

**Fig. 5:**
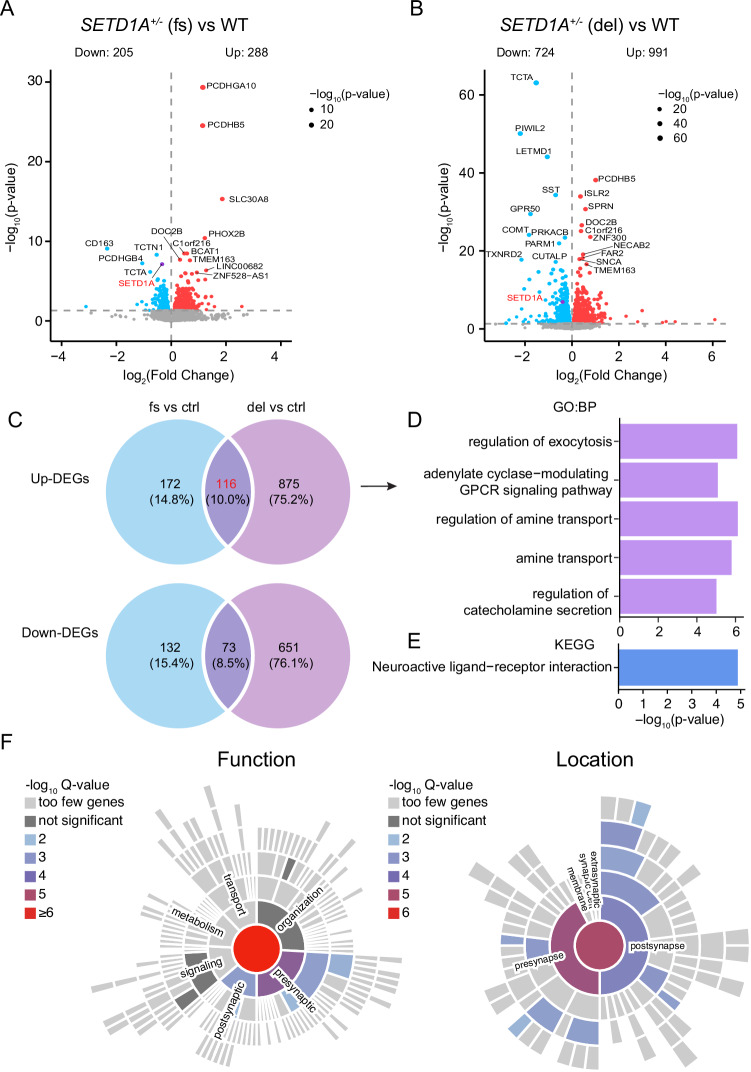
Transcriptomic analysis of Ngn2-iNs carrying *SETD1A*^*+/−*^ LoF mutations. **A**, **B** Volcano plot of Ngn2-iNs carrying c.4596_4597insG and c.4582-2delAG mutations compared to control. Differential expression is defined by adjusted p-value < 0.05 and log_2_FC > 0. **C** Venn diagram shows the overlapped DEGs between c.4582-2delAG and c.4582-2delAG mutations. **D**–**F** The enriched GO biological pathways (**D**), enriched KEGG terms (**E**) and Syn-go function and location enrichment analysis (**F**) of overlapped 116 up-regulated DEGs.

To understand how *SETD1A*^*+/−*^ LoF mutations (c.4596_4597insG and c.4582-2delAG) altered synaptic function in human iN cells, we conducted a transcriptomic analysis using bulk RNA sequencing (RNA seq) in 6-week-old iNs of both control and mutant neurons. Our RNA seq results confirmed the absence of chromosomal abnormality in all 5 isogenic pairs (Fig. S5) by eSNP-Karyotyping. We then examined the expression level of *SETD1A*. Consistent with our qPCR data (Fig. 2A), we found that *SETD1A* is significantly downregulated in *SETD1A*^*+/−*^ LoF mutant human iN cells (Figs. 5A, B, S7A). Along with the changes of *SETD1A*, we identified a substantial number of differentially expressed genes (DEGs) (493 DEGs for c.4596_4597insG and 1705 DEGs for c.4582-2delAG) in *SETD1A* mutant neurons when compared with control human iN cells (Fig. 5A, B).

To understand the biological pathways and functions of the dysregulated DEGs, we conducted Gene Ontology (GO) enrichment analyses. For *SETD1A* c.4596_4597insG mutation, the up-regulated genes (n = 288) showed significant enrichment for synaptic function and amine transport-associated pathways; however, the down-regulated genes (n = 205) did not show any significantly enriched pathways (Fig. S6A). For *SETD1A* c.4582-2delAG mutation, the up-regulated genes (n = 991) are also enriched in synaptic function and amine transport-associated biological pathways (Fig. S6B). In addition, the down-regulated genes (n = 724) are also enriched for axon development and axon guidance-related pathways (Fig. S6C). Interestingly, we also found that several metabolic pathways, such as glycolysis and Adenosine Diphosphate (ADP) metabolic process, are enriched in the up-regulated DEGs in c.4582-2delAG dataset, suggesting a wide-spread effect of *SETD1A*^*+/−*^ LoF mutation that may be beyond direct neuron-related function.

Given the similar effects of the patient-specific mutation c.4582-2delAG and the other exon-16 mutation c.4596_4597insG that mimics c.4582-2delAG, we further narrowed down the DEGs by examining the shared DEGs in these two datasets (Fig. 5C). We identified 116 shared up-regulated DEGs and 73 shared down-regulated DEGs (Fig. 5C). Consistent with previous GO analysis results, GO enrichment analysis revealed that the overlapped up-regulated genes are enriched for biological pathways related to regulation of exocytosis and amine transport (Fig. 5D, E), which highlights the impact of *SETD1A*^*+/−*^ LoF on synaptic function. To further understand the function of these overlapped 116 up-regulated DEGs, we did Synaptic Gene Ontologies (Syn-GO) analysis [48] and found genes associated with synaptic function are significantly enriched in the presynaptic terminal (Fig. 5F). To further gain a gene network view of these DEGs, we conducted protein-protein-interaction (PPI) analysis using Search Tool for the Retrieval of Interacting Genes (STRING) [49]. The PPI network of 116 overlapped up-regulated DEGs was constructed with a medium confidence score (0.4) based on both functional and physical protein associations. We found significant enrichments of several biological processes, including regulation of amine transport, postsynaptic membrane potential, chemical transmission, and exocytosis (Fig. S7B). The PPI analysis of cellular component showed proteins are enriched mainly in the axon terminus, pre-, and post-synaptic membrane (Fig. S7C). These results largely support that dendritic arborization is altered in *SETD1A*^*+/−*^ LoF human iN cells, and synaptic transmission is enhanced (mEPSCs frequency and recovery of the readily releasable pool of the synaptic vesicles) (Fig. 4).

**Fig. 6:**
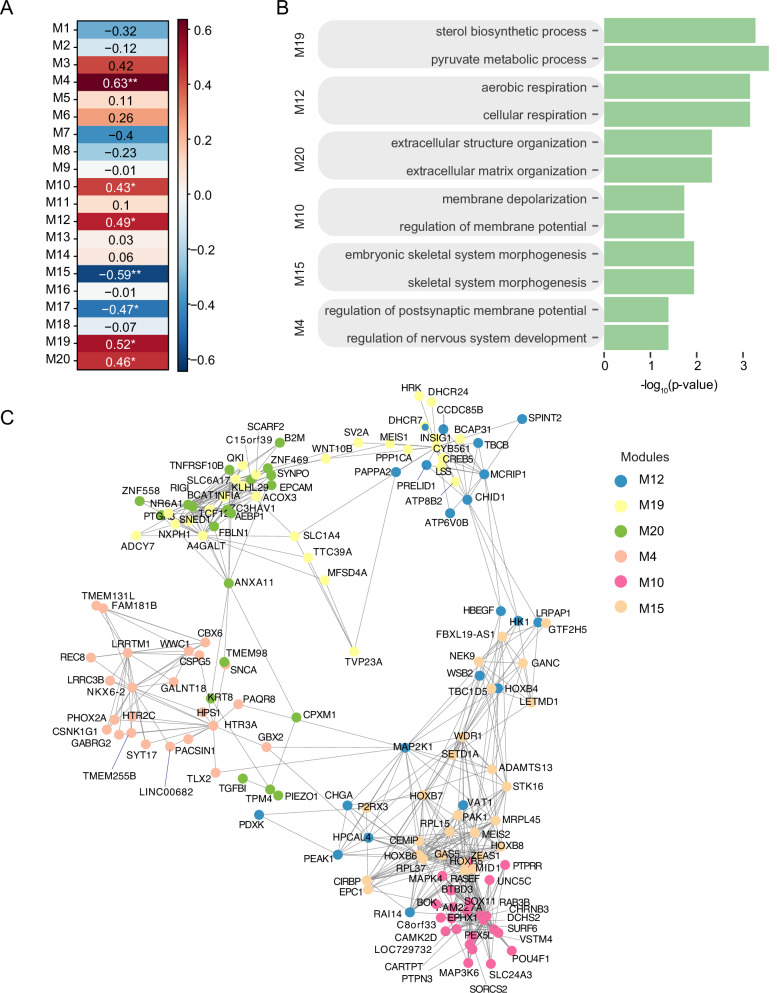
WGCNA analysis of Ngn2-iNs carrying *SETD1A*^*+/−*^ LoF mutations. **A** Heatmap shows the correlation between the identified 20 modules and phenotype (*SETD1A*^*+/−*^ LoF mutations). **B** The enriched GO biological pathways for modules are significantly associated with *SETD1A*^*+/−*^ LoF mutations. **C** The gene co-expression networks in 6 modules are visualized by Cytoscape.

To further understand the regulated transcriptional network, we constructed gene networks to identify modules of highly co-expressed genes, a powerful method to identify biological processes in an unsupervised manner [50, 51]. We conducted weighted gene co-expression network analysis (WGCNA) analysis [35] by combining the c.4596_4597insG and c.4582-2delAG mutation datasets together. Here, we identified 20 modules in total with seven of them showing a significant correlation with *SETD1A*^*+/−*^ LoF mutation (Fig. 6A). To understand the biological pathways enriched in these seven modules, we conducted GO analysis for genes in each module (Fig. 6B). We found that six out of the seven *SETD1A*^*+/−*^ LoF mutation-associated modules showed significant enrichment for biological pathways, such as metabolic pathways (M19 and M12), development of nervous system (M4), synaptic function and membrane potential regulation (M10), extracellular matrix organization (M20), and skeletal system morphogenesis (M15) (Fig. 6B). In addition, gene co-expression networks of 6 *SETD1A*^*+/−*^ LoF mutation-associated modules are visualized using Cytoscape [37] (Fig. 6C). Interestingly, *SETD1A* was included in M15, a module showing significant association with *SETD1A*^*+/−*^ LoF mutation, indicating *SETD1A* as a critical regulator of gene co-expressions in M15. These results suggest that human iNs carrying the *SETD1A*^*+/−*^ LoF mutations have altered gene co-expression networks that are mainly enriched in cell metabolism, synaptic function, and development of nervous system, underscoring the specific impact of *SETD1A*^*+/−*^ LoF on human neuronal function, metabolism, and development.

## Discussion

Using human neuronal cells derived from five isogenic pairs of iPSCs carrying the patient-specific c.4582-2delAG and c.4596_4597insG (p.Leu1533fs) mutations in *SETD1A* gene, we investigated the impact of *SETD1A*^*+/−*^ LoF on neuronal transcriptional profiling, morphology, and functions. We found that the patient mutation c.4582-2delAG in *SETD1A* causes *SETD1A* haploinsufficiency and dysregulates synapse functionality. We found that both c.4582-2delAG and c.4596_4597insG cause a reduction of SETD1A full-length protein expression, which is accompanied by a reduction in *SETD1A* mRNA expression levels. Morphological analysis revealed that *SETD1A*^*+/−*^ LoF affects dendritic development; electrophysiological analysis revealed an altered synaptic transmission in human iNs carrying *SETD1A*^*+/−*^ LoF mutations; transcriptional analysis identified DEGs that are associated with dendritic morphogenesis and synaptic transmission. Our findings indicate that patient-specific *SETD1A* mutation (i.e., c.4582-2delAG) dysregulates synaptic function.

Large-scale human genetics studies in SCZ have identified that SCZ cases carry a significantly higher burden of rare premature ORF termination variants among evolutionarily constrained genes across different ancestries [2, 52]. The interpretation of the effects of the rare premature ORF termination variants is important for the accurate determination of variant impacts. It has been shown that NMD effects are highly concordant across tissues and individuals, which may at least partially address the impact of premature ORF termination variants in humans [53]. Interindividual differences of RNA stability may account for the heterogenous phenotypical expression of the disease phenotype in humans [54]. Based on previous findings, however, SCZ-associated *OTUD7A* LoF causes cellular phenotype without NMD in human neurons [55], suggesting that NMD is not the only hypothesis related to SCZ premature ORF termination variants. Our analyses indicated that both c.4582-2delAG and c.4596_4597insG mutations lead to the reduction of *SETD1A* mRNA and protein levels. The patient-specific c.4582-2delAG mutation is associated not only with SCZ but also neurodevelopmental disorders (NDDs) [7]. Our results indicate that *SETD1A* mRNA reduction can be restored directly through inhibiting NMD by CHX in human iPSCs. It is highly suggested that NMD plays a role in regulating *SETD1A* mRNAs and protein expressions in humans. Since there are many different types of mutations identified in *SETD1A* [7], further studies are required to precisely interpret different mutations accounting for the heterogeneous genetic causes.

Our morphological results demonstrate that *SETD1A*^*+/−*^ LoF mutations decrease dendrite complexity without affecting synapse formation and dendrite length. This is largely consistent with previous studies using both mouse and human neuron culture models for *SETD1A*^*+/−*^ LoF [17–21]. Three studies of *Setd1a* haploinsufficiency in mouse models revealed deficits in dendrite development and decreased dendritic spine density [17–19]. However, human neuronal studies about excitatory and inhibitory neurons in vitro showed increased dendrite length at a later time point without synaptic puncta formation changes [20]. Another cortical spheroid model showed decreased neurite outgrowth in cortical neurons [21]. Overall, both in vivo and in vitro studies converge in altered dendrite complexity as a common morphological phenotype for *SETD1A*^*+/−*^ LoF. Although the detailed mechanism remains unclear, our transcriptomic studies revealed that the expression of genes associated with axon development is downregulated by *SETD1A*^*+/−*^ LoF.

The synaptic hypothesis of SCZ has been proposed to be the underlying convergent biological pathway that contributes to the pathophysiology of SCZ [56]. Recent GWAS studies also support synaptic dysregulation as a major biological process associated with SCZ and other NDDs [3]. Using human neurons as a model system, our study demonstrates that *SETD1A*^*+/−*^ LoF mutations lead to synaptic dysregulation, including enhanced synaptic transmission, enhanced neuronal excitability, and altered synaptic plasticity. The increased miniature excitatory synaptic transmission and enhanced neuronal excitability suggest hyperactivity in excitatory neurons and possible increased release probability in the presynaptic compartments resulting from *SETD1A*^*+/−*^ LoF. However, our evoked recording results indicate that there is no difference in AMPAR-mediated EPSC and significantly increased PPR in neurons carrying *SETD1A*^*+/−*^ LoF, suggesting potential decreased releasing probability in presynaptic terminals. This apparent paradoxical phenotype could be explained by an increased recovery of readily releasable pools after each release in the *SETD1A* mutant human neurons. Interestingly, a presynaptic protein DOC2B showed a consistent increase in mRNA expression levels from the RNA seq data of the *SETD1A* mutant human neurons (Fig. 4O). It has been shown that DOC2B is an important modulator for spontaneous synaptic release and asynchronous synaptic release [47, 57–59]. The knockdown of DOC2 families leads to decreased frequency of spontaneous release without affecting evoked release, and it appears that DOC2 protein in the presynaptic compartment regulates spontaneous release in a Ca^2+^-independent manner [47]. Although the detailed mechanisms remain to be elucidated, the observed altered synaptic transmission and synaptic plasticity are also supported by mouse prefrontal cortex slice recording [17, 18], indicating synaptic dysregulation as a common phenotype caused by *SETD1A*^*+/−*^ LoF.

Transcriptomic analysis reveals up-regulation of synaptic transmission, ion transport, and cell metabolism-related biological pathways and down-regulation of axon development and guidance. These data support an enhanced synaptic function and defective dendritic development in human neurons that we found in this study. This is also consistent with the previously reported gene expression data in both mouse and human neuron studies [17, 20]. When the DEGs from the *SETD1A*^*+/−*^ LoF neurons were compared with DEGs with the chromatin immunoprecipitation sequencing (ChIP-seq) database of SETD1A from mouse prefrontal cortex [17], we found 58 overlapping DEGs and 18 overlapping DEGs for c.4582-2delAG and c.4596_4597insG (p.Leu1533fs), respectively. Since SETD1A is involved in histone post-translational regulations that may affect gene expression [17, 19, 20], future studies should be carried out to test whether the epigenetic landscape is affected by *SETD1A*^*+/−*^ LoF in human neurons.

This study on *SETD1A*^*+/−*^ LoF in the human neuronal system also has limitations. It mainly focuses on studying the impact of *SETD1A*^*+/−*^ LoF in induced human excitatory neurons, which transdifferentiates iPSCs to induced excitatory neurons by ectopic expression of transcription factor Ngn2 [22]. This model may not fully capture the differentiation progress that occurs during the neurodevelopmental stage, as our study solely focuses on excitatory neurons. It’s known that SETD1A is broadly expressed in all brain cell types, including other neuronal subtypes such as inhibitory neurons, dopaminergic neurons, and glial cells. The cell-type-specific impact of *SETD1A*^*+/−*^ LoF has been supported by two mouse studies [17, 19]. Therefore, it is important to study *SETD1A* function in different models and different cell types. For example, disease-relevant mRNA NMD might be cell-lineage specific, it is important to clarify in future experiments whether *SETD1A* LoF mutation impact on *SETD1A* NMD is cell-type, or –lineage specific. Nevertheless, since many mutations identified in *SETD1A* are associated with SCZ and NDDs, it is important to study the mutation-specific impact of *SETD1A* related to different diseases.

Despite methodological differences, the consistent observation across studies is that *SETD1A*^*+/−*^ LoF mutations significantly impact dendritic architecture and synaptic function. This convergence suggests that SETD1A plays a crucial role in maintaining neuronal structural integrity and synaptic function. Future multi-modality functional assay of neurons carrying *SETD1A* LoF mutations especially in the context of other neurodevelopmental disorder risk genes on a large scale will further establish the functional role of *SETD1A* in contributing to neuropsychiatric and neurodevelopmental disorders [60]. Given the association of various *SETD1A* mutations with different NDDs and SCZ, it is evident that understanding the biological functions of SETD1A in different neural contexts is essential for unraveling the pathophysiology of these conditions. Our study highlights the crucial role of SETD1A in neuronal development and synaptic function, with implications for understanding the pathophysiology of SCZ and NDDs. Further research is needed to elucidate the precise mechanisms and develop targeted therapies.

## Supplementary information

Supplemental Information

Dataset 1

Dataset 2

## Data Availability

The scripts for running the RNA-Seq processing and eSNP-karyotyping pipeline are provided at https://zenodo.org/records/13840811. Raw and processed RNA-Seq data were deposited and available on the NCBI Gene Expression Omnibus under accession number GSE278808.

## Code availability

The scripts for running the RNA-Seq processing and eSNP-karyotyping pipeline are provided at https://zenodo.org/records/13840811. Raw and processed RNA-Seq data were deposited and available on the NCBI Gene Expression Omnibus under accession number GSE278808.

## References

[1] Owen MJ, Sawa A, Mortensen PB. Schizophrenia. Lancet. 2016;388:86–97. 26777917 10.1016/S0140-6736(15)01121-6 PMC4940219

[2] Singh T, Poterba T, Curtis D, Akil H, Al Eissa M, Barchas JD, et al. Rare coding variants in ten genes confer substantial risk for schizophrenia. Nature. 2022;604:509–16. 35396579 10.1038/s41586-022-04556-w PMC9805802

[3] Trubetskoy V, Pardinas AF, Qi T, Panagiotaropoulou G, Awasthi S, Bigdeli TB, et al. Mapping genomic loci implicates genes and synaptic biology in schizophrenia. Nature. 2022;604:502–8. 35396580 10.1038/s41586-022-04434-5 PMC9392466

[4] Palmer DS, Howrigan DP, Chapman SB, Adolfsson R, Bass N, Blackwood D, et al. Exome sequencing in bipolar disorder identifies AKAP11 as a risk gene shared with schizophrenia. Nat Genet. 2022;54:541–7. 35410376 10.1038/s41588-022-01034-x PMC9117467

[5] Takata A, Xu B, Ionita-Laza I, Roos JL, Gogos JA, Karayiorgou M. Loss-of-function variants in schizophrenia risk and SETD1A as a candidate susceptibility gene. Neuron. 2014;82:773–80. 24853937 10.1016/j.neuron.2014.04.043 PMC4387883

[6] Guipponi M, Santoni FA, Setola V, Gehrig C, Rotharmel M, Cuenca M, et al. Exome sequencing in 53 sporadic cases of schizophrenia identifies 18 putative candidate genes. PLoS One. 2014;9:e112745. 25420024 10.1371/journal.pone.0112745 PMC4242613

[7] Singh T, Kurki MI, Curtis D, Purcell SM, Crooks L, McRae J, et al. Rare loss-of-function variants in SETD1A are associated with schizophrenia and developmental disorders. Nat Neurosci. 2016;19:571–7. 26974950 10.1038/nn.4267 PMC6689268

[8] Kummeling J, Stremmelaar DE, Raun N, Reijnders MRF, Willemsen MH, Ruiterkamp-Versteeg M, et al. Characterization of SETD1A haploinsufficiency in humans and Drosophila defines a novel neurodevelopmental syndrome. Mol Psychiatry. 2021;26:2013–24. 32346159 10.1038/s41380-020-0725-5

[9] Yu X, Yang L, Li J, Li W, Li D, Wang R, et al. De novo and inherited SETD1A variants in early-onset epilepsy. Neurosci Bull. 2019;35:1045–57. 31197650 10.1007/s12264-019-00400-w PMC6864154

[10] Lan M, Wang Y, Li S, Zhao L, Liu P, Hu W. Case report: de novo variant of SETD1A causes infantile epileptic spasms syndrome. Front Neurol. 2023;14:1278035. 37928142 10.3389/fneur.2023.1278035 PMC10620521

[11] Yuen CRK, Merico D, Bookman M, Howe LJ, Thiruvahindrapuram B, Patel RV, et al. Whole genome sequencing resource identifies 18 new candidate genes for autism spectrum disorder. Nat Neurosci. 2017;20:602–11. 28263302 10.1038/nn.4524 PMC5501701

[12] Feliciano P, Zhou X, Astrovskaya I, Turner TN, Wang T, Brueggeman L, et al. Exome sequencing of 457 autism families recruited online provides evidence for autism risk genes. NPJ Genom Med. 2019;4:19. 31452935 10.1038/s41525-019-0093-8 PMC6707204

[13] Shilatifard A. The COMPASS family of histone H3K4 methylases: mechanisms of regulation in development and disease pathogenesis. Annu Rev Biochem. 2012;81:65–95. 22663077 10.1146/annurev-biochem-051710-134100 PMC4010150

[14] Network, Pathway Analysis Subgroup of Psychiatric Genomics C. Psychiatric Genome-Wide Association study analyses implicate neuronal, immune and histone pathways. Nat Neurosci. 2015;18:199–209. 25599223 10.1038/nn.3922 PMC4378867

[15] Girdhar K, Hoffman GE, Jiang Y, Brown L, Kundakovic M, Hauberg ME, et al. Cell-specific histone modification maps in the human frontal lobe link schizophrenia risk to the neuronal epigenome. Nat Neurosci. 2018;21:1126–36. 30038276 10.1038/s41593-018-0187-0 PMC6063773

[16] Hoshii T, Cifani P, Feng Z, Huang CH, Koche R, Chen CW, et al. A non-catalytic function of SETD1A regulates cyclin K and the DNA damage response. Cell. 2018;172:1007–21.e1017. 29474905 10.1016/j.cell.2018.01.032 PMC6052445

[17] Mukai J, Cannavo E, Crabtree GW, Sun Z, Diamantopoulou A, Thakur P, et al. Recapitulation and reversal of schizophrenia-related phenotypes in Setd1a-deficient mice. Neuron. 2019;104:471–87.e412. 31606247 10.1016/j.neuron.2019.09.014 PMC7010348

[18] Nagahama K, Sakoori K, Watanabe T, Kishi Y, Kawaji K, Koebis M, et al. Setd1a insufficiency in mice attenuates excitatory synaptic function and recapitulates schizophrenia-related behavioral abnormalities. Cell Rep. 2020;32:108126. 32937141 10.1016/j.celrep.2020.108126

[19] Chen R, Liu Y, Djekidel MN, Chen W, Bhattacherjee A, Chen Z, et al. Cell type-specific mechanism of Setd1a heterozygosity in schizophrenia pathogenesis. Sci Adv. 2022;8:eabm1077. 35245111 10.1126/sciadv.abm1077 PMC8896793

[20] Wang S, Rhijn JV, Akkouh I, Kogo N, Maas N, Bleeck A, et al. Loss-of-function variants in the schizophrenia risk gene SETD1A alter neuronal network activity in human neurons through the cAMP/PKA pathway. Cell Rep. 2022;39:110790. 35508131 10.1016/j.celrep.2022.110790 PMC7615788

[21] Chong ZS, Khong ZJ, Tay SH, Ng SY. Metabolic contributions to neuronal deficits caused by genomic disruption of schizophrenia risk gene SETD1A. Schizophrenia (Heidelb). 2022;8:115. 36581615 10.1038/s41537-022-00326-9 PMC9800576

[22] Zhang Y, Pak C, Han Y, Ahlenius H, Zhang Z, Chanda S, et al. Rapid single-step induction of functional neurons from human pluripotent stem cells. Neuron. 2013;78:785–98. 23764284 10.1016/j.neuron.2013.05.029 PMC3751803

[23] Levinson DF, Duan J, Oh S, Wang K, Sanders AR, Shi J, et al. Copy number variants in schizophrenia: confirmation of five previous findings and new evidence for 3q29 microdeletions and VIPR2 duplications. Am J Psychiatry. 2011;168:302–16. 21285140 10.1176/appi.ajp.2010.10060876 PMC4441324

[24] Zhang S, Zhang H, Zhou Y, Qiao M, Zhao S, Kozlova A, et al. Allele-specific open chromatin in human iPSC neurons elucidates functional disease variants. Science. 2020;369:561–5. 32732423 10.1126/science.aay3983 PMC7773145

[25] Zhang S, Zhang H, Forrest MP, Zhou Y, Sun X, Bagchi VA, et al. Multiple genes in a single GWAS risk locus synergistically mediate aberrant synaptic development and function in human neurons. Cell Genom. 2023;3:100399. 37719141 10.1016/j.xgen.2023.100399 PMC10504676

[26] Muller FJ, Brandl B, Loring JF. Assessment of human pluripotent stem cells with PluriTest. Cambridge (MA): StemBook; 2008. 23658970

[27] Kang Y, Zhou Y, Li Y, Han Y, Xu J, Niu W, et al. A human forebrain organoid model of fragile X syndrome exhibits altered neurogenesis and highlights new treatment strategies. Nat Neurosci. 2021;24:1377–91. 34413513 10.1038/s41593-021-00913-6 PMC8484073

[28] Halikere A, Popova D, Scarnati MS, Hamod A, Swerdel MR, Moore JC, et al. Addiction associated N40D mu-opioid receptor variant modulates synaptic function in human neurons. Mol Psychiatry. 2020;25:1406–19. 31481756 10.1038/s41380-019-0507-0 PMC7051890

[29] Ran FA, Hsu PD, Wright J, Agarwala V, Scott DA, Zhang F. Genome engineering using the CRISPR-Cas9 system. Nat Protoc. 2013;8:2281–308. 24157548 10.1038/nprot.2013.143 PMC3969860

[30] Pang ZP, Yang N, Vierbuchen T, Ostermeier A, Fuentes DR, Yang TQ, et al. Induction of human neuronal cells by defined transcription factors. Nature. 2011;476:220–3. 21617644 10.1038/nature10202 PMC3159048

[31] Fantuzzo JA, Mirabella VR, Hamod AH, Hart RP, Zahn JD, Pang ZP Intellicount: high-throughput quantification of fluorescent synaptic protein puncta by machine learning. eNeuro 2017;4. 10.1523/ENEURO.0219-17.2017. 10.1523/ENEURO.0219-17.2017 PMC5718246 29218324

[32] Pang ZP, Xu W, Cao P, Sudhof TC. Calmodulin suppresses synaptotagmin-2 transcription in cortical neurons. J Biol Chem. 2010;285:33930–9. 20729199 10.1074/jbc.M110.150151 PMC2962493

[33] Kim D, Paggi JM, Park C, Bennett C, Salzberg SL. Graph-based genome alignment and genotyping with HISAT2 and HISAT-genotype. Nat Biotechnol. 2019;37:907–15. 31375807 10.1038/s41587-019-0201-4 PMC7605509

[34] Zhang Y, Parmigiani G, Johnson WE. ComBat-seq: batch effect adjustment for RNA-seq count data. NAR Genom Bioinform. 2020;2:lqaa078. 33015620 10.1093/nargab/lqaa078 PMC7518324

[35] Langfelder P, Horvath S. WGCNA: an R package for weighted correlation network analysis. BMC Bioinformatics. 2008;9:559. 19114008 10.1186/1471-2105-9-559 PMC2631488

[36] Yu G, Wang LG, Han Y, He QY. clusterProfiler: an R package for comparing biological themes among gene clusters. OMICS. 2012;16:284–7. 22455463 10.1089/omi.2011.0118 PMC3339379

[37] Shannon P, Markiel A, Ozier O, Baliga NS, Wang JT, Ramage D, et al. Cytoscape: a software environment for integrated models of biomolecular interaction networks. Genome Res. 2003;13:2498–504. 14597658 10.1101/gr.1239303 PMC403769

[38] Di Tommaso P, Chatzou M, Floden EW, Barja PP, Palumbo E, Notredame C. Nextflow enables reproducible computational workflows. Nat Biotechnol. 2017;35:316–9. 28398311 10.1038/nbt.3820

[39] Patro R, Duggal G, Love MI, Irizarry RA, Kingsford C. Salmon provides fast and bias-aware quantification of transcript expression. Nat Methods. 2017;14:417–9. 28263959 10.1038/nmeth.4197 PMC5600148

[40] Li H, Handsaker B, Wysoker A, Fennell T, Ruan J, Homer N, et al. The Sequence Alignment/Map format and SAMtools. Bioinformatics. 2009;25:2078–9. 19505943 10.1093/bioinformatics/btp352 PMC2723002

[41] Weissbein U, Schachter M, Egli D, Benvenisty N. Analysis of chromosomal aberrations and recombination by allelic bias in RNA-Seq. Nat Commun. 2016;7:12144. 27385103 10.1038/ncomms12144 PMC4941052

[42] Sherry ST, Ward MH, Kholodov M, Baker J, Phan L, Smigielski EM, et al. dbSNP: the NCBI database of genetic variation. Nucleic Acids Res. 2001;29:308–11. 11125122 10.1093/nar/29.1.308 PMC29783

[43] Hallson G, Hollebakken RE, Li T, Syrzycka M, Kim I, Cotsworth S, et al. dSet1 is the main H3K4 di- and tri-methyltransferase throughout Drosophila development. Genetics. 2012;190:91–100. 22048023 10.1534/genetics.111.135863 PMC3249358

[44] Lykke-Andersen S, Jensen TH. Nonsense-mediated mRNA decay: an intricate machinery that shapes transcriptomes. Nat Rev Mol Cell Biol. 2015;16:665–77. 26397022 10.1038/nrm4063

[45] Nehme R, Zuccaro E, Ghosh SD, Li C, Sherwood JL, Pietilainen O, et al. Combining NGN2 programming with developmental patterning generates human excitatory neurons with NMDAR-mediated synaptic transmission. Cell Rep. 2018;23:2509–23. 29791859 10.1016/j.celrep.2018.04.066 PMC6003669

[46] Maximov A, Pang ZP, Tervo DG, Sudhof TC. Monitoring synaptic transmission in primary neuronal cultures using local extracellular stimulation. J Neurosci Methods. 2007;161:75–87. 17118459 10.1016/j.jneumeth.2006.10.009

[47] Pang ZP, Bacaj T, Yang X, Zhou P, Xu W, Sudhof TC. Doc2 supports spontaneous synaptic transmission by a Ca(2+)-independent mechanism. Neuron. 2011;70:244–51. 21521611 10.1016/j.neuron.2011.03.011 PMC3102832

[48] Koopmans F, van Nierop P, Andres-Alonso M, Byrnes A, Cijsouw T, Coba MP, et al. SynGO: an evidence-based, expert-curated knowledge base for the synapse. Neuron. 2019;103:217–34.e214. 31171447 10.1016/j.neuron.2019.05.002 PMC6764089

[49] Szklarczyk D, Gable AL, Nastou KC, Lyon D, Kirsch R, Pyysalo S, et al. The STRING database in 2021: customizable protein-protein networks, and functional characterization of user-uploaded gene/measurement sets. Nucleic Acids Res. 2021;49:D605–D12. 33237311 10.1093/nar/gkaa1074 PMC7779004

[50] Parikshak NN, Gandal MJ, Geschwind DH. Systems biology and gene networks in neurodevelopmental and neurodegenerative disorders. Nat Rev Genet. 2015;16:441–58. 26149713 10.1038/nrg3934 PMC4699316

[51] Li J, Zhou D, Qiu W, Shi Y, Yang JJ, Chen S, et al. Application of weighted gene Co-expression network analysis for data from paired design. Sci Rep. 2018;8:622. 29330528 10.1038/s41598-017-18705-z PMC5766625

[52] Liu D, Meyer D, Fennessy B, Feng C, Cheng E, Johnson JS, et al. Schizophrenia risk conferred by rare protein-truncating variants is conserved across diverse human populations. Nat Genet. 2023;55:369–76. 36914870 10.1038/s41588-023-01305-1 PMC10011128

[53] Teran NA, Nachun DC, Eulalio T, Ferraro NM, Smail C, Rivas MA, et al. Nonsense-mediated decay is highly stable across individuals and tissues. Am J Hum Genet. 2021;108:1401–8. 34216550 10.1016/j.ajhg.2021.06.008 PMC8387471

[54] Duan J, Shi J, Ge X, Dolken L, Moy W, He D, et al. Genome-wide survey of interindividual differences of RNA stability in human lymphoblastoid cell lines. Sci Rep. 2013;3:1318. 23422947 10.1038/srep01318 PMC3576867

[55] Kozlova A, Zhang S, Kotlar AV, Jamison B, Zhang H, Shi S, et al. Loss of function of OTUD7A in the schizophrenia- associated 15q13.3 deletion impairs synapse development and function in human neurons. Am J Hum Genet. 2022;109:1500–19. 35931052 10.1016/j.ajhg.2022.07.001 PMC9388388

[56] Howes OD, Onwordi EC. The synaptic hypothesis of schizophrenia version III: a master mechanism. Mol Psychiatry. 2023;28:1843–56. 37041418 10.1038/s41380-023-02043-w PMC10575788

[57] Xue R, Gaffaney JD, Chapman ER. Structural elements that underlie Doc2beta function during asynchronous synaptic transmission. Proc Natl Acad Sci USA. 2015;112:E4316–25. 26195798 10.1073/pnas.1502288112 PMC4534243

[58] Groffen AJ, Martens S, Diez Arazola R, Cornelisse LN, Lozovaya N, de Jong AP, et al. Doc2b is a high-affinity Ca^2+^ sensor for spontaneous neurotransmitter release. Science. 2010;327:1614–8. 20150444 10.1126/science.1183765 PMC2846320

[59] Yao J, Gaffaney JD, Kwon SE, Chapman ER. Doc2 is a Ca^2+^ sensor required for asynchronous neurotransmitter release. Cell. 2011;147:666–77. 22036572 10.1016/j.cell.2011.09.046 PMC3220409

[60] Zhang H, McCarroll A, Peyton L, Diaz de Leon-Guerrerro S, Zhang S, Gowda P, et al. Scaled and efficient derivation of loss-of-function alleles in risk genes for neurodevelopmental and psychiatric disorders in human iPSCs. Stem Cell Reports. 2024;19:1489–504. 39270650 10.1016/j.stemcr.2024.08.003 PMC11561461

