# Supplementary material for: Mutations of schizophrenia risk gene SETD1A dysregulate synaptic function in human neurons
Source: Mol Psychiatry. 2025 Sep 17;30(12):5680–93. doi: 10.1038/s41380-025-03246-z (PMC12602330; doi:10.1038/s41380-025-03246-z)
Supplement: Supplementary file 1 — Supplemental Information [file 41380_2025_3246_MOESM1_ESM.docx]

Xiao Su et al. **Supplemental Information**

**Schizophrenia patient-specific mutations in SETD1A cause mRNA nonsense-mediated decay and functional impairment**

**Supplementary Table 1: The primers used for CRISPR gene editing**

| SETD1A_ssODN_altered_PAM | CGCCGCCGCGTCCTCCTGCCACTCACTTCCCTGCCCTGCTCACCTCCTCCCTGCCGTGTGTCTCACGGGACGAATCGCGTGCTGTCCGAGCGCCGGTCCGAGCAGCGGCGGCTGCTGAGCGCCATCGGT |
| --- | --- |
| SETD1A onTar gRNA | ACCGGCGCTCGGACAGCACG |
| SETD1A on-target Primer F | TGGCCAGAGAGGAGCCGTTCTC |
| SETD1A on-target Primer R | AGACACCTCCCACACCCCAGAC |
| SETD1A off-target Primer F-1 | GGGACACAGGAAAACATGGT |
| SETD1A off-target Primer R-1 | TGAGATTTTGTCATCGCCCG |
| SETD1A off-target Primer F-2 | ATTCTGAAAGGGTACGCAGC |
| SETD1A off-target Primer R-2 | CAACACCCATCGTCACTGTG |
| SETD1A off-target Primer F-3 | TTCTCTGAAGCTCCTCTCGG |
| SETD1A off-target Primer R-3 | TAAACGGGCTCTCTGAACCT |
| SETD1A off-target Primer F-4 | CACCTCCGATCACCCCTG |
| SETD1A off-target Primer R-4 | CAGAATCAGGGGTGCCAATG |
| SETD1A off-target Primer F-5 | TATGCTCTACCACGTCTCGC |
| SETD1A off-target Primer R-5 | GAAATGCTAGCACCACACCA |

**Supplementary Table 2: Primers used for qPCR**

| SETD1A exon 4-5 forward primer | GAAACGGTCAAAAACCTCCA |
| --- | --- |
| SETD1A exon 4-5 reverse primer | CGAGCCTTGGAACTTCTCAC |
| SETD1A exon 17-18 forward primer | CTGACGAGATGGTCATCGAA |
| SETD1A exon 17-18 reverse primer | TGCAGCAGTGGTTGATGAAT |


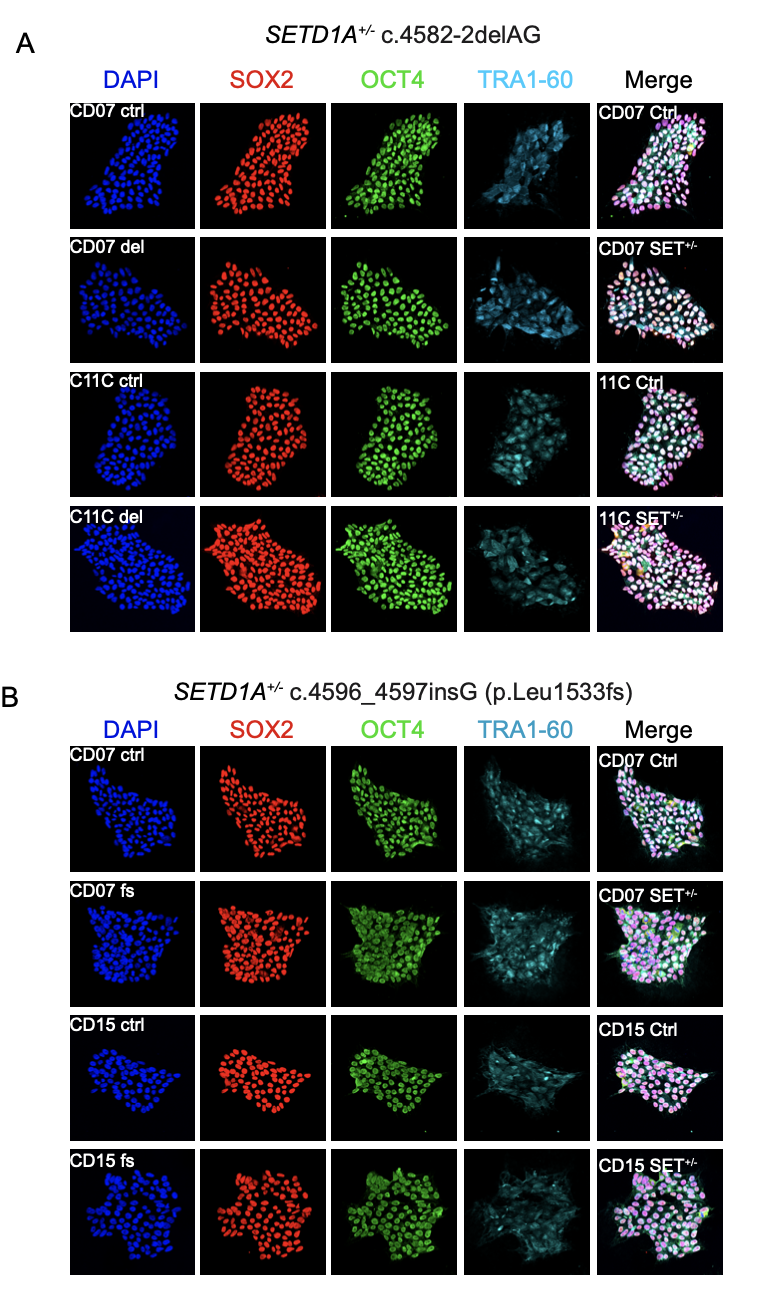


**Supplementary Figure 1: Pluripotency marker of engineered iPSCs.** (**A**) The representative images of two isogenic pairs of c.4582-2delAG immunostained with pluripotency marker SOX2, OCT4, TRA1-60 and nuclei marker DAPI. (**B**) The representative images of two isogenic pairs of c.4596_4597insG (p. Leu1533fs) immunostained with pluripotency marker SOX2, OCT4, TRA1-60 and nuclei marker DAPI.


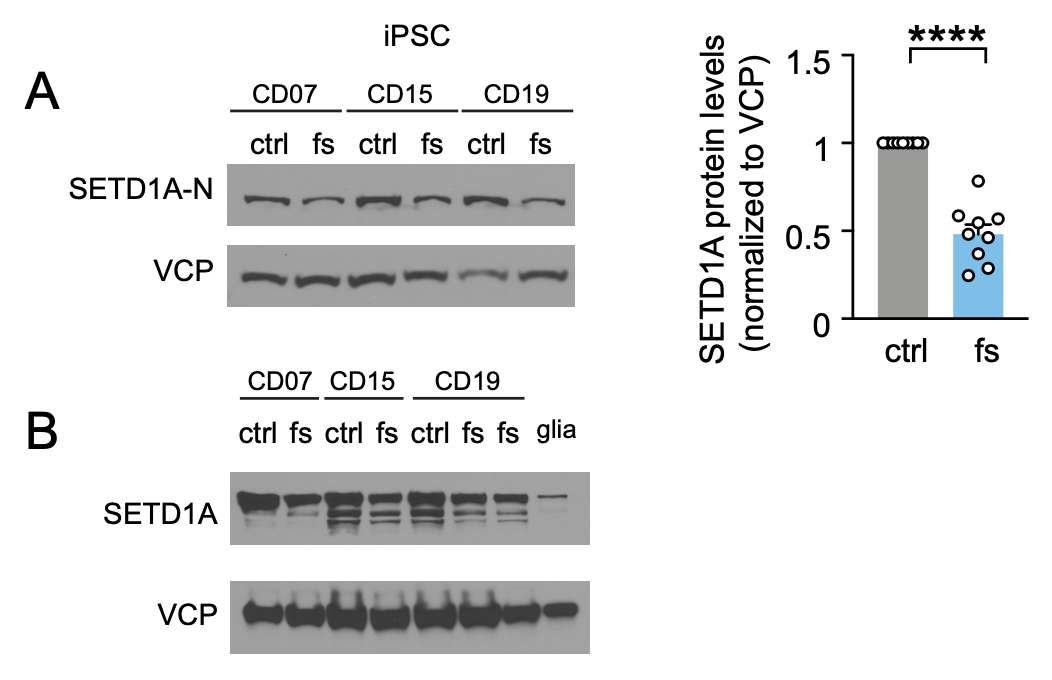


**Supplementary Figure 2: Western blot of iPSCs and Ngn2-iNs carrying *SETD1A^+/-^* c.4596_4597insG mutation.** (**A**) Western blot of SETD1A (N-terminal) protein normalized to VCP in iPSCs. N=9 for p.L1533fs mutation (3 independent batches of 3 isogenic pairs). (**B**) The representative western blot of SETD1A expression in primary glia culture. Statistical significance was assessed by unpaired t-test. Data are shown as means ± SEM. *P < 0.05, **P < 0.01, ***P < 0.001 and **** P < 0.0001.


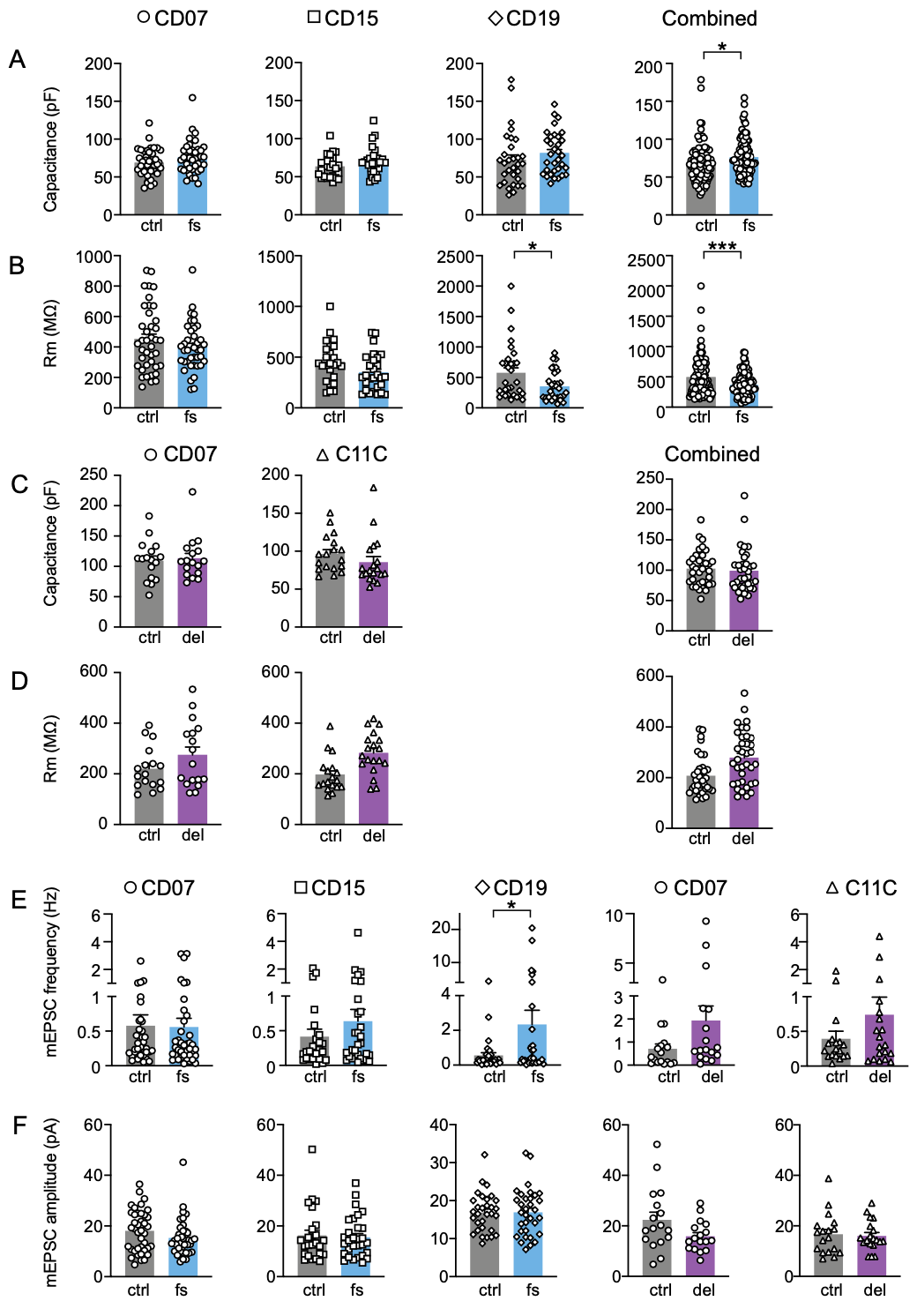


**Supplementary Figure 3: Electrophysiological parameters of Ngn2-iNs carrying** **c.4596_4597insG and c.4582-2delAG mutation for each isogenic pair.** (**A&B**) The analysis of cell membrane capacitance (**A**) and membrane resistance (**B**) for c.4596_4597insG mutation. (**C&D**) The analysis of cell membrane capacitance (**C**) and membrane resistance (**D**) for c.4582-2delAG mutation. (**E&F**) The analysis of mEPSC frequency (**E**) and amplitude (**F**) for each isogenic pair. Statistical significance was assessed by unpaired t-test. Data are shown as means ± SEM. *P < 0.05, **P < 0.01, ***P < 0.001 and **** P < 0.0001.


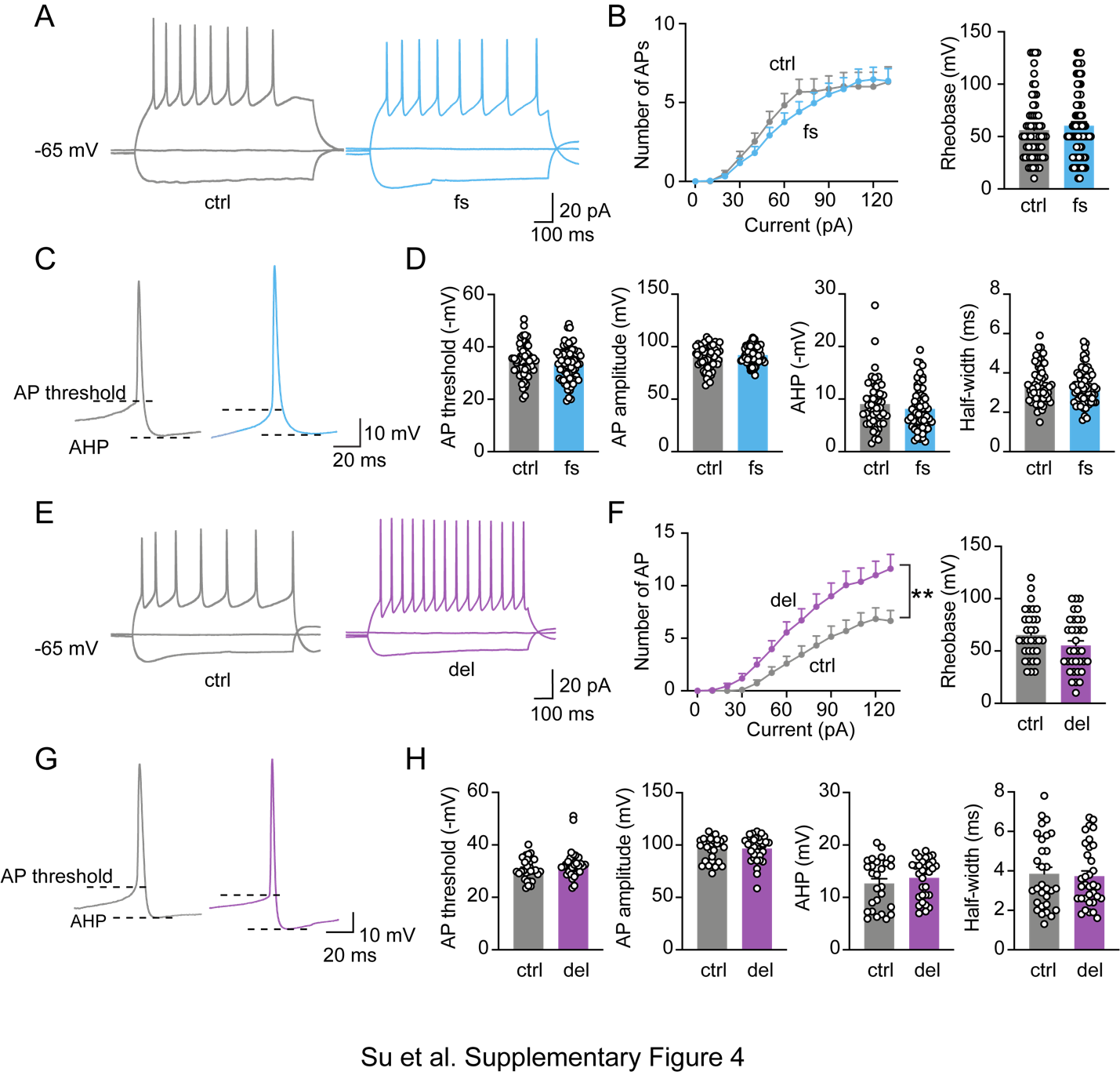


**Supplementary Figure 4: The characterization of neuronal excitability of Ngn2-iNs carrying** **c.4596_4597insG and c.4582-2delAG mutation.** (**A**) The representative traces of evoked AP for c.4596_4597insG mutation. (**B**) The analysis of evoked AP and rheobase for c.4596_4597insG mutation. (**C**) The representative traces of first evoked AP for c.4596_4597insG mutation. (**D**)The analysis of first evoked AP including AP threshold, AP amplitude, afterhyperpolarization (AHP) and half-width. (**E**) The representative traces of evoked AP for c.4582-2delAG mutation. (**F**) The analysis of evoked AP and rheobase for c.4582-2delAG mutation. (**G**) The representative traces of first evoked AP for c.4582-2delAG mutation. (**H**)The analysis of first evoked AP including AP threshold, AP amplitude, afterhyperpolarization (AHP) and half-width. Statistical significance was assessed by unpaired t-test. Data are shown as means ± SEM. *P < 0.05, **P < 0.01, ***P < 0.001 and **** P < 0.0001.


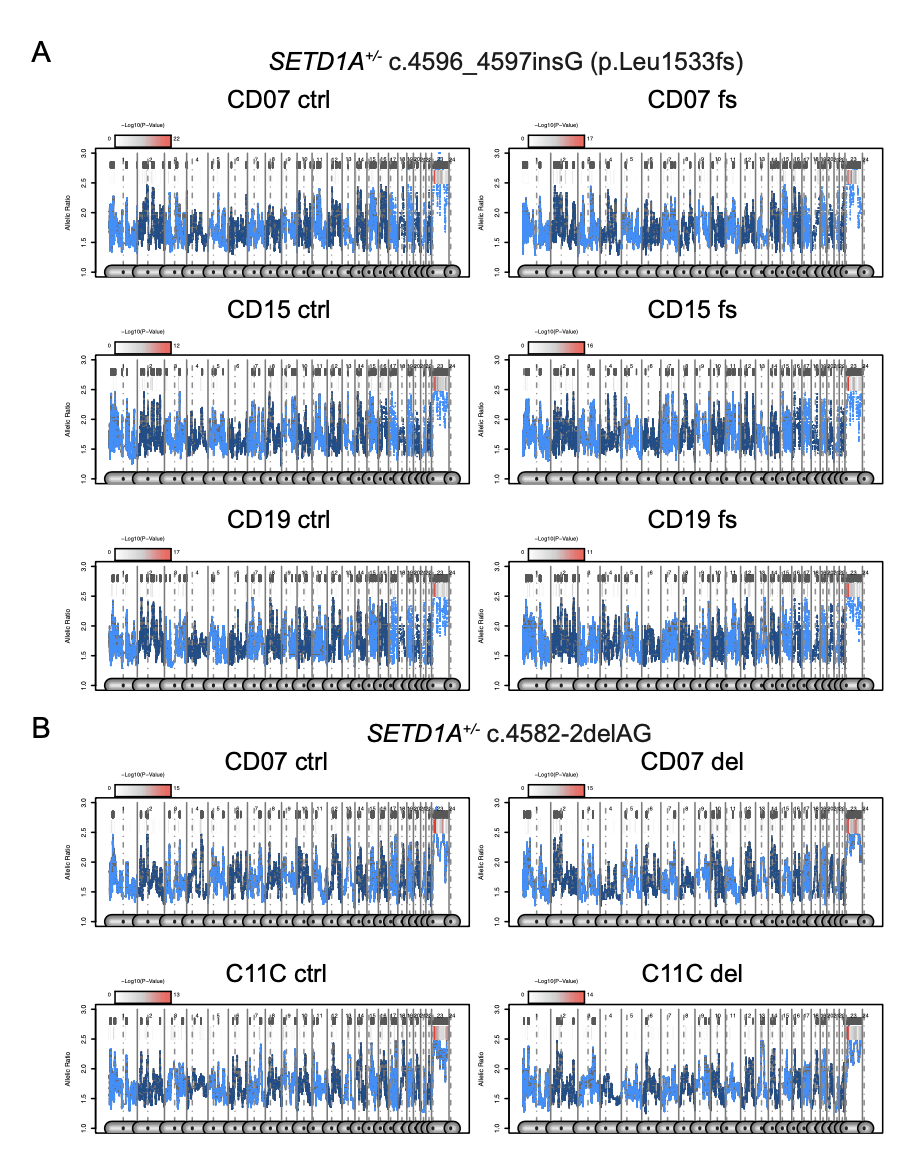


**Supplementary Figure 5: RNA-seq-based eSNP-Karyotyping analysis of Ngn2-iNs carrying** **c.4596_4597insG and c.4582-2delAG mutation. (A&B)** Each panel showed the moving average of SNP intensity (RNA-seq reads) of the two alleles of heterozygous SNPs. Only the allelic ratio graphs of batch edited lines are shown here; Note that only an autosomal peak interval marked with a red bar above the peak is considered to be significantly “abnormal”, and no iPSC lines showed such abnormality for c.4596_4597insG and c.4582-2delAG mutation.


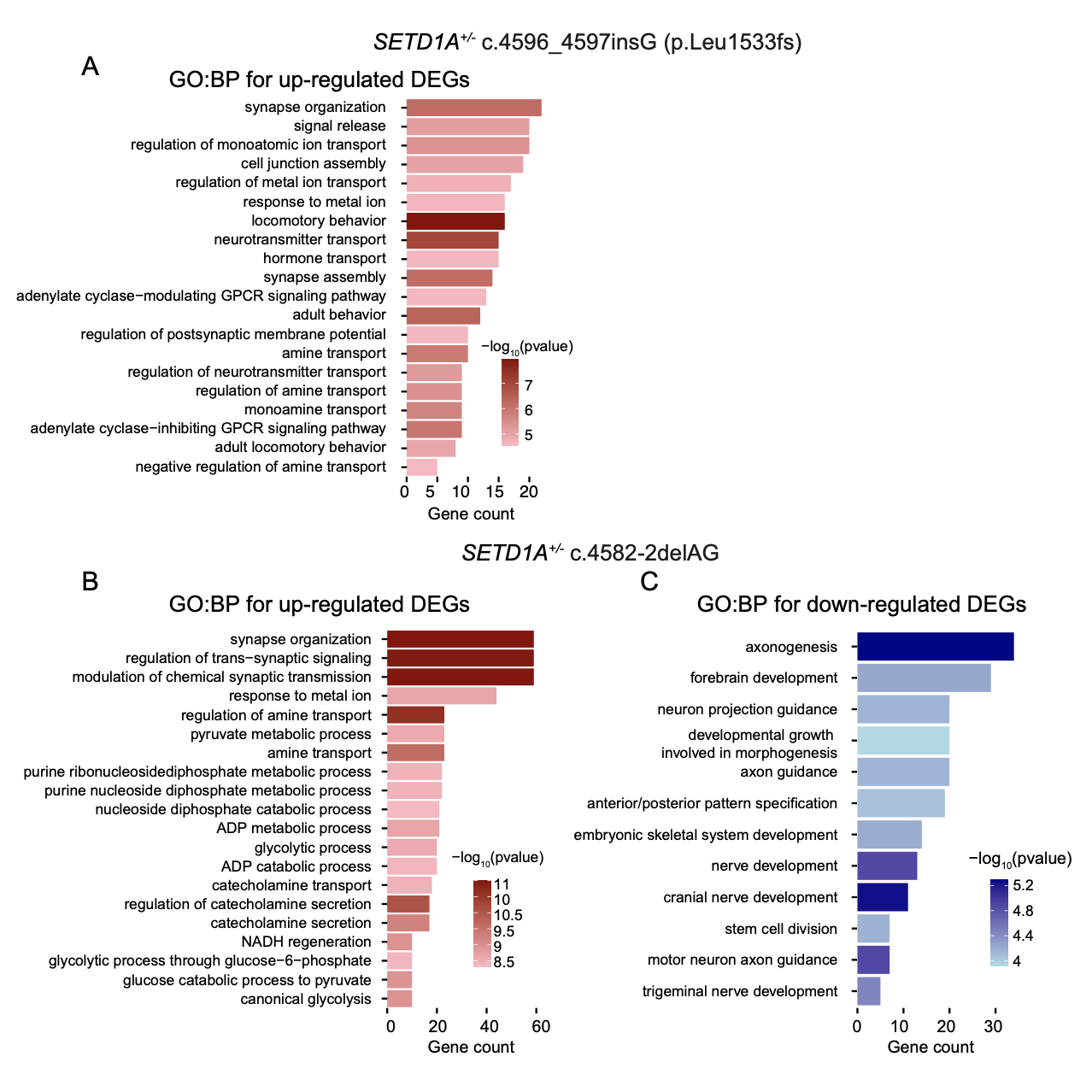


**Supplementary Figure 6: The GO analysis of Ngn2-iNs carrying** **c.4596_4597insG and c.4582-2delAG mutation.** (**A**) GO analysis of up-regulated DEGs in RNA seq data for c.4596_4597insG mutation. (**B&C**) GO analysis of up-regulated and down-regulated DEGs in RNA seq data for c.4582-2delAG mutation.


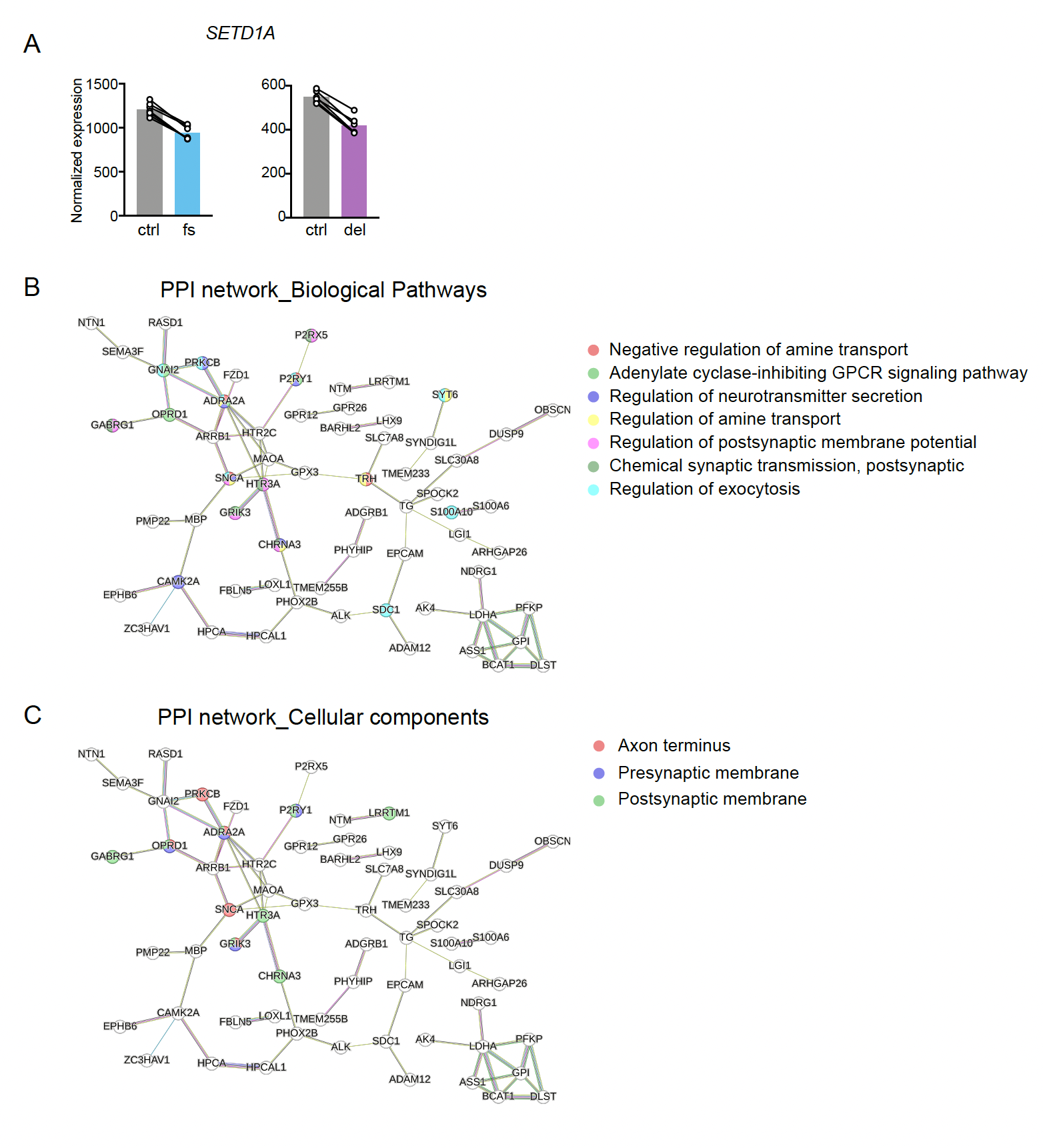


**Supplementary Figure 7: The gene expression of *SETD1A* and *DOC2B* and PPI network analysis.** (**A**) Normalized expression of *SETD1A* in RNA seq data for c.4596_4597insG and c.4582-2delAG mutation. (**B&C**) PPI network analysis for overlapped up-regulated DEGs in biological pathways and cellular components. Top 7 enriched biological pathways and top 3 enriched cellular components are shown here.
